# A cumulative meta‐analysis of the effects of individual physical activity interventions targeting healthy adults

**DOI:** 10.1111/obr.12690

**Published:** 2018-04-27

**Authors:** R. Love, J. Adams, E. M. F. van Sluijs, C. Foster, D. Humphreys

**Affiliations:** ^1^ Department of Social Policy and Intervention University of Oxford Oxford UK; ^2^ Centre for Diet and Activity Research (CEDAR), MRC Epidemiology Unit University of Cambridge Cambridge UK; ^3^ Centre for Exercise, Nutrition and Health Sciences University of Bristol Bristol UK

**Keywords:** Cumulative analysis, physical activity, scientific progress, systematic review

## Abstract

Despite a large and increasing evidence base on physical activity interventions, the high rates of physical inactivity and associated chronic diseases are continuing to increase globally. The purpose of this cumulative meta‐analysis was to investigate the evolution of randomized controlled trial evidence of individual‐level physical activity interventions to asses if new trials are contributing novel evidence to the field. Through a two‐staged search process, primary studies examining the effects of interventions targeted at increasing physical activity within healthy adult populations were pooled and selected from eligible systematic reviews. Cumulative meta‐analyses were performed on effect sizes immediately post‐intervention (n = 62), and for long‐term behaviour change (≥12‐month post‐baseline; n = 27). Sufficiency and stability of the evidence was assessed through application of pre‐published indicators. Meta‐analyses suggest overall positive intervention effects on physical activity. The evidence base for effectiveness immediately post‐intervention reached levels of sufficiency and stability in 2007; and for long‐term follow‐up in 2011. In the time since, intervention effectiveness has not substantially changed, and further trials are unlikely to change the direction and magnitude of effect. Substantial evidence exists demonstrating that physical activity interventions can modify individual behaviour in controlled settings. Researchers are urged to shift focus towards investigating the optimization, implementation, sustainability and cost‐effectiveness of interventions.

## Introduction

Physical inactivity is the fourth leading risk factor for global mortality 1. Worldwide, 31.1% of adults are physically inactive, which is projected to cause 5.3 million of the 57 million deaths worldwide, accounting for 9% of premature mortality and 6–10% of all deaths from major non‐communicable diseases 2. It is estimated that a decrease in population level physical inactivity by only 10% would prevent half a million deaths each year 3. A recent analysis of 130,000 individuals revealed across 17 high, middle and low income countries higher levels of physical activity are consistently associated with a lower risk of mortality and cardiovascular disease 4. Alongside physical inactivity being an independent risk factor for cardiovascular disease, it further compounds the mortality risk through its association with obesity 5. Thus, increasing physical activity offers a simple and low‐cost strategy of tackling rising rates of both obesity and chronic disease globally. In response to mounting evidence of these associations, the World Health Organization targets for physical activity, together with the United Nation's goals on non‐communicable disease, have led to increasing adoption of national policies and recognition for the promotion of physical activity as a key element of efforts to improve the health of populations 6. Accordingly, over the last two decades, a wide range of interventions have been developed and employed in efforts to improve population levels of physical activity. Alongside, a large scientific literature base of evaluations of physical activity interventions has amassed 7. Despite this growing intervention literature together with increased public health efforts, evidence updated in 2016 indicates that worldwide figures of physical inactivity are not improving 8.

Ideally, the results of early intervention evaluations inform the development and design of future interventions and through the accumulation of evidence, knowledge and innovation lead to the production of more effective interventions. However, successful promotion of physical activity across populations has lagged relative to available evidence 9. Despite the large literature base and consistently increasing number of published trials, the field is failing to influence behaviour at a population level. Researchers have attributed this to failures in both the scale‐up of effective interventions and the management of the uncertainty of how to optimize physical activity interventions 8, 10. Thus, it is an opportune time to reflect on the accumulation of evidence and consider how current efforts can be improved.

As new interventions are developed, evaluated and published, evidence evolves over time, with new knowledge strengthening and updating, or altering and invalidating the results documented in earlier trials 11. Cumulative meta‐analyses provide a framework to describe trends in summary estimates of effects over time and identify the benefits of interventions as early as possible 12. The approach was first utilized in 1992 for assessment of trials for myocardial infarction. In the time since, it has been applied to numerous public health interventions, including a recent analysis of signage‐based stair use interventions 13, 14. By recalculating the aggregate effect estimate each time a study is published, the approach allows determination of the point at which additional data is unlikely to change the conclusion. Here, we present the results of a cumulative meta‐analysis of adult physical activity promotion interventions to investigate the evolution of randomized controlled trial (RCT)‐based evidence in this field, and use an approach relevant to public health to examine whether and when sufficiency and stability of evidence has been achieved for the evidence of effective short‐term and long‐term physical activity behaviour change 13.

## Methods

A two‐staged selection strategy was used to gather evidence. First, relevant systematic reviews were identified through a systematic search. Secondly, primary studies were pooled from selected systematic reviews, screened against the inclusion criteria and relevant trials selected. Physical activity data from included trials was then cumulatively meta‐analysed. Screening, data extraction and risk of bias assessments were completed by the primary author, with quality checks performed by a second author. The review was directed by the preferred reporting items for systematic reviews and meta‐analyses guidelines (PRISMA).

### Search strategy and selection criteria

In stage one, six electronic databases (The Cochrane Central Register of Controlled Trials, MEDLINE/PubMed, EMBASE, CINAHL, PsycINFO and Web of Science) were searched for relevant systematic reviews. Searches were conducted in May 2015 with pre‐piloted search strategies (Data S1). Systematic reviews that examined the effects of interventions targeted at healthy adults to promote physical activity and included RCTs, were considered for inclusion. Reviews that did not utilize a systematic process for identifying studies were excluded. In stage two, primary studies were extracted from the included systematic reviews, and all RCTs in healthy adults, comparing the effect of a physical activity promotion intervention to a control condition, considered for inclusion. Included trials were restricted to those reporting a continuous measure of physical activity, and reporting N (sample size), mean and standard deviation (SD) for the intervention and control group at follow‐up. As a result of potential problems with cross‐over and wait‐list trials, including spillover effects from the waiting period to intervention phase, and loss of a viable control group making comparisons impossible, only data up to the point of cross‐over was extracted and included in the analysis. Resulting from the heterogeneous characteristics of interventions targeted at obese populations and individuals with pre‐existing medical conditions, inclusion was restricted to interventions aimed at increasing physical activity within a generally healthy adult population. Inclusion was further limited to interventions with a continuous measure of individual duration of physical activity measured either objectively or subjectively (e.g. via accelerometer and questionnaire) at baseline and follow‐up. In order to develop a homogeneous set of data for analysis, trials with alternative indicators of activity (e.g. motivation to exercise) or sedentary behaviour were not included. A detailed overview of the inclusion and exclusion criteria for both stages is outlined in Table 1.

**Table 1:** Description of inclusion and exclusion criteria for Phases 1 (systematic review) and 2 (primary study) selection process

	Included	Excluded
**Phase 1: systematic reviews**		
Population	Reviews that included studies targeted at a general adult population (between 16 and 65 years)	Reviews with a central focus on the inclusion of studies targeted at participants with a medical condition
Intervention	Randomized controlled trials (RCTs) were eligible for inclusion in the review	
Study design	Reviews following an identified systematic process	Narrative and other non‐systematic reviews that did not outline a systematic process for identifying and synthesizing studies
**Phase 2: primary studies**		
Population	Targeted at the general adult population with participants with a mean age greater then 16, less than 65 years	Targeted at children (<16 years) and elderly individuals (>65 years)
	Participants must have been free from pre‐existing medical conditions or with no more than 10% of subjects with pre‐existing medical conditions	Study population with greater than 10% of subjects with pre‐existing medical conditions
		Trials where the mean baseline BMI (kg/m^2^) was above 30 (obese BMI classes)
Intervention	All physical activity interventions explicitly aimed at promoting change in the behaviour of participants at the individual level	Environmental changes, policy approaches and mass media campaigns
Study design	RCTs in which individuals were allocated individually or by cluster	All non‐randomized designs
	Active intervention arms must have been compared with a control arm (standard or usual care) or wait list control condition	All qualitative studies
		RCTs only comparing two active intervention
Outcomes	Reported continuous measure of physical activity with at least one time‐point post‐baseline	Those that did not report subjectively or objectively measured physical activity as a continuous, outcome measure
Publication type	Peer reviewed journal article	Conference abstract, study protocol, report, dissertation, book
Publication year	Any year	N/A
Publication language	English	Any other language

BMI, body mass index; RCTs, randomized controlled trial.

### Data analysis

Intervention characteristics were extracted for each primary study meeting the inclusion criteria. Effect size data was extracted at the first follow‐up post intervention, and (where available) for long‐term behaviour change (defined as at least 12‐month post‐baseline). Post‐baseline, as opposed to post‐intervention, time‐points were included to assess the effect on long‐term behaviour change, irrespective of intervention length. If multiple long‐term follow‐up measures were reported, these were collapsed into a single intervention effect. When adequate data was not reported in primary studies, where available, we extracted this from published reviews that had utilized author data requests to clarify outcome values 10, 15, 16. For trials in which multiple intervention arms were presented compared with a single comparison group, the conditions were collapsed into a single mean intervention effect through calculation of a pooled mean and SD (as outlined in section 7.7.3.8 of the Cochrane Handbook) 17.

Intervention effects for outcome measures were calculated and expressed as standardized mean differences (SMDs), based on Hedges's adjusted *g* and its 95% confidence intervals (95% CI) 18. Because included studies measured physical activity across a variety of scales, SMD, calculated as the observed difference in means relative to an estimate of the SD, was used. Hedge's *g* was selected as the index of mean difference as it is the preferred approach when the majority of included studies have small sample sizes with comparably greater standard errors 19.

To assess differences in the accumulation of evidence for behaviour change immediately following an intervention trial and long‐term behaviour change, two separate cumulative meta‐analyses were conducted. The first, intervention effect at the follow‐up time point closest to intervention end, included all trials in the review. The second included only those trials reporting assessments at least 12‐month post‐baseline. The cumulative meta‐analysis provides cumulative pooled estimates and 95% CIs. As studies are successively added, the overall SMD and 95% CIs are recalculated providing evidence of the evolution of intervention effects over time. To assess the sequential contributions of trials and evaluate changes in effectiveness over time, studies were added alphabetically by year of publication to a random‐effects models using the *metacum* user written command in STATA version 14.0.

Random effects models, based on the method of moments, were used under the assumption that the true effect sizes estimated by individual studies were drawn from a distribution of true effects rather than a single value, and an expectation of substantial heterogeneity given the differences in interventions eligible for inclusion 20. Given that random effect models can overestimate intervention effects in comparison to fixed effect models, a comparison of models can reveal the presence of small study effects that may result from publication or other biases. As both fixed (Mantel Haenszel) and random effects (DerSimonian and Laird) models produced comparable results, all analyses are presented using random effects estimates.

To address the question of whether and when sufficient evidence had been accumulated that the addition of further trials would not change established conclusions, Muellerleile's indicators of sufficiency and stability were applied to both cumulative meta‐analyses 13. As defined by Muellerleile, sufficiency of the evidence was determined through evaluation of the failsafe ratio as each new trial was added to the cumulative meta‐analysis. The failsafe ratio is a measure of the number of trials with null results required to make the meta‐analytic result non‐statistically significant (failsafe number), versus 5× (+10) the number of trials already available (Rosenthal standard). A failsafe ratio exceeding 1 indicates that there is sufficient evidence that additional research is unlikely to change the existing conclusion. Stability was assessed by calculating the cumulative slope of the regression line of the cumulative meta‐analysis over time; when this becomes less than 0·005, it is suggested that the combined evidence has reached stability.

## Results

**Figure 1:**
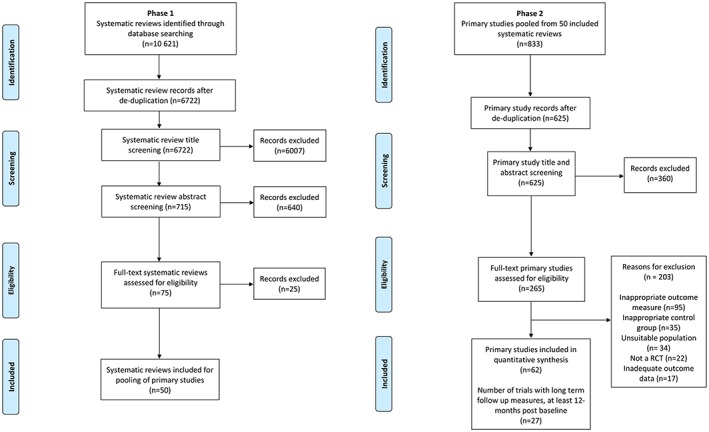
Study selection. [Colour figure can be viewed at http://wileyonlinelibrary.com]

Figure 1 shows the trial identification process, which identified 62 unique trials published up to 2013 (Data S2 and S3 provide reference lists of included and excluded studies). Of the 62 unique trials, 27 provided adequate data for inclusion in the long‐term behaviour change analysis.

A summary of intervention characteristics is outlined Table 2. The mean participant age in studies was 47.2 years (SD: 9.5) and 67% of participants were female. The included trials took place predominately in home (48%) and primary care (27%) settings, with fewer implemented in community (15%) and workplace settings (10%). Interventions were delivered predominately on an individual basis (77.4%) as opposed to through group settings (17.7%). As outlined, interventions were spread across various modes of delivery (virtual, in‐person). Subjective forms of physical activity measurement (primarily through questionnaire) was utilized substantially more than objective measurement (77% vs. 23%).

**Table 2:** Characteristics of included studies

	No.	(%)		No.	(%)
Country			Delivery format		
United States	37	59.7	Virtual	22	35.5
United Kingdom	8	12.9	In person	19	30.6
Netherlands	5	8.0	Both	21	33.9
Australia or New Zealand	6	9.7	Delivery method		
Other	6	9.7	Individual	48	77.4
Setting of intervention			Group setting	11	17.7
Community	9	14.5	Frequency of intervention contact		
Home	30	48.4	Monthly or more	19	30.6
Primary care	17	27.4	Repeated less then monthly	30	48.4
Workplace	6	9.7	Once only	13	21.0
Measurement tool			Follow‐up time		
Objective (e.g. accelerometer)	14	22.6	Greater than 6 months	41	66.1
Subjective (e.g. questionnaire)	48	77.4	Less than 6 months	21	33.9
Continuous characteristics	Mean	SD		Mean	SD
Participant percentage male	32.8	26.7	Intervention duration	21.1	16.9
Mean age	47.2	9.5	Follow‐up time (weeks)	26.4	21.6

SD, standard deviation.

**Figure 2:**
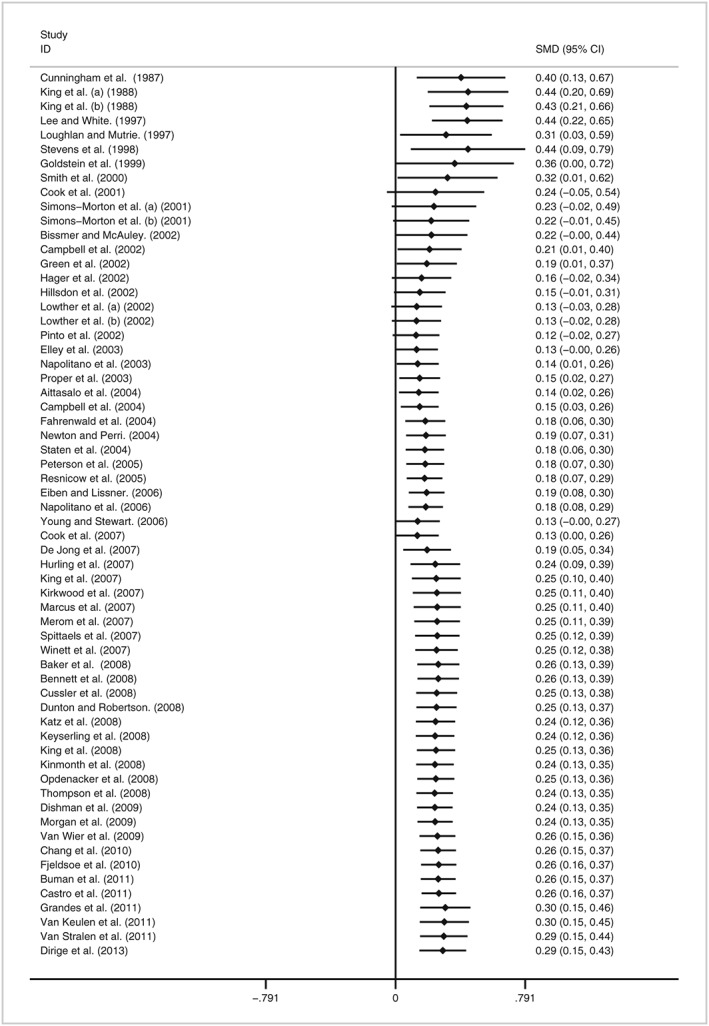
Cumulative meta‐analysis of intervention effect immediately post‐intervention.

Figure 2 shows the cumulative meta‐analysis of post‐intervention effect sizes (*n* = 62). Early trials conducted throughout the 1980s and 1990s demonstrated a high degree of heterogeneity, with the first published results revealing statistically significant, positive effects. However, the conduct of additional trials weakened the initial evidence of effectiveness, with the overall effect no longer being significant by the ninth trial. Two trials in 1997 and 1999 reporting harmful, negative intervention effects contributed to this decrease in effect size. The overall effect regains statistical significance following the addition of the 33rd trial in 2007. According to the pre‐established thresholds (Data S4), a sufficient and stable body of evidence with the potential to change the physical activity behaviour of participants immediately following intervention implementation, was achieved in 2007 with the addition of the 39th trial. Since then, we identified 23 further RCTs were reported up untill to 2013.

**Figure 3:**
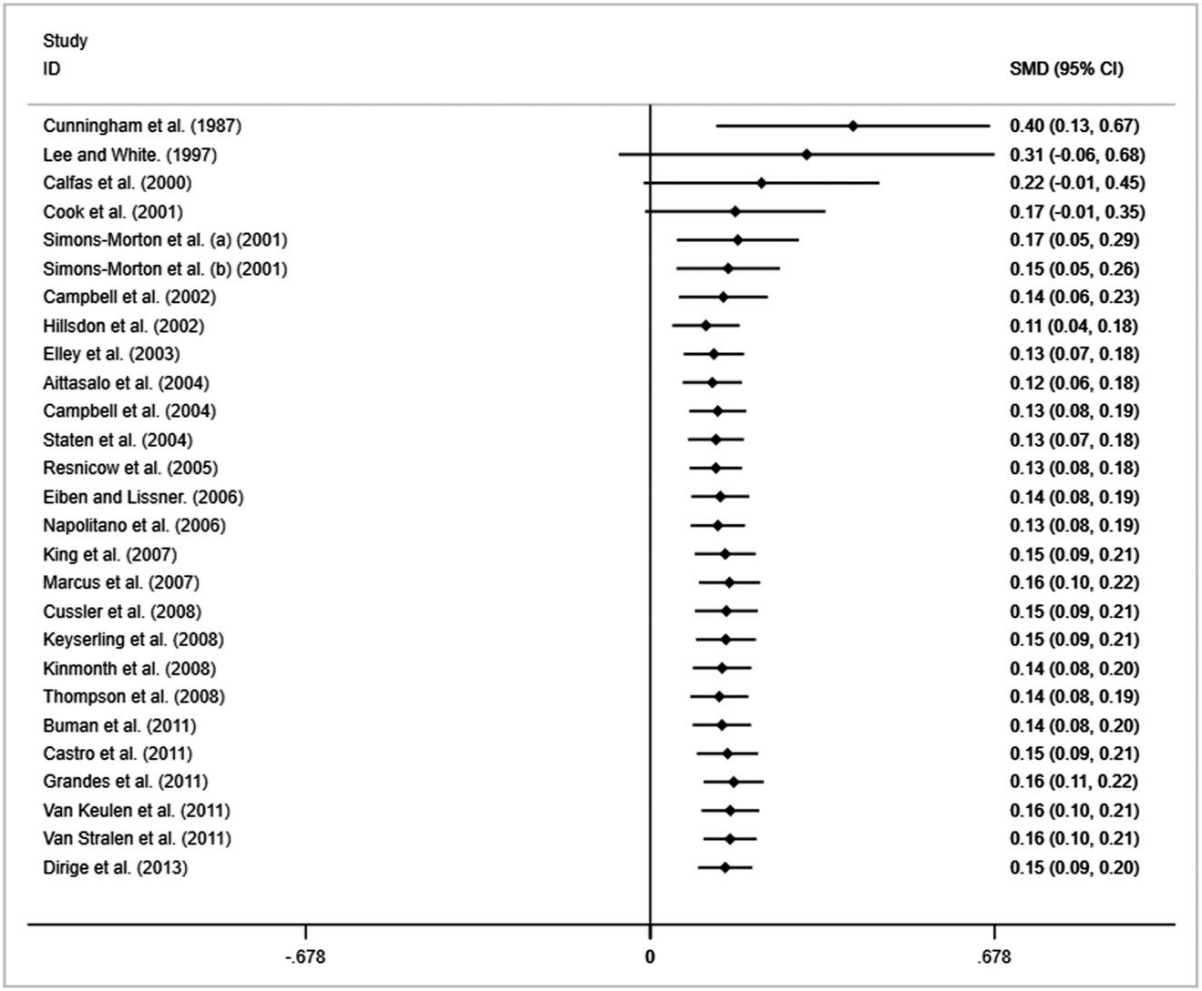
Cumulative meta‐analysis of intervention effect on long‐term behaviour change (trials with follow‐up at least 12‐month post‐baseline).

When the analysis was restricted to interventions with physical activity measurements at least 12‐month post‐baseline (*n* = 27), the thresholds of sufficiency and stability were not met until later. As displayed in Figure 3, similarly to the post‐intervention analysis, early effects demonstrated large amounts of heterogeneity with a steadily decreasing effect as subsequent interventions are added. While a statistically significant overall effect was achieved in 2001, the combined thresholds of sufficiency and stability are not met until 2011 with the addition of the 23rd trial (Data S5). Only four further trials assessing long‐term behaviour change had been reported after this point.

## Discussion

This cumulative meta‐analysis of individual‐level physical activity interventions demonstrates that we have strong randomized evidence that physical activity levels can be improved and maintained. Estimates of post‐intervention effects and long‐term behaviour change have not changed substantially since 2007 and 2011, respectively, and additional trials are increasingly unlikely to change these stabilized findings. In the time following the attainment of predetermined thresholds of sufficiency and stability for post‐intervention effects, we identified 23 further RCTs published to 2013. Although not included in the review, further intervention trials have been conducted and published since we ran our searches 21, 22, 23.

To our knowledge, this is the first cumulative meta‐analysis of physical activity interventions. Overall, the results question the need for further trials testing the short‐term effectiveness of individual physical activity interventions in healthy adult populations in highly controlled settings. The attainment of thresholds of sufficiency and stability, yet lack of impact on a population scale, indicate a need for a shift in research focus from controlled effectiveness trials to the optimization of interventions that effectively maintain behaviour change over the long term, within real world settings. Our results reinforce prior calls for a shift from the repetition of individual physical activity trials to focus on the sustained effects of interventions in practice 8. This should include adequate consideration for cost‐effectiveness to enable identification of interventions that achieve maximum population health benefits relative to cost 24.

To build knowledge regarding how to optimize interventions to achieve long‐term behaviour change within populations is a need for critical consideration of the decision making processes around what we evaluate. In theory, the decision to develop a trial to evaluate a new physical activity promotion intervention is determined by the ability of the trial to add value and change current knowledge and practice 25. Thus, ideally, new trials should build on prior evidence and through a cumulative process lead to the development of more effective interventions. However, evidence suggests that the norm is to reinvent programs and approaches, rather than directly building and innovating on previous findings 26.

Continuing to test interventions against standard, no treatment control conditions, provides little information about the relative effects of different interventions and intervention components, restricting the ability to build on prior knowledge. Given the sufficiency and stability of evidence within highly controlled settings, yet rising population levels of physical inactivity, there needs to be a shift towards developing evaluations to help us understand what works. Traditional two‐arm RCTS assess the effectiveness of the whole intervention versus control, without an ability to determine which intervention components and settings are, or are not, contributing to the effect. This evaluative approach continues to persist; 80% of RCTs registered between 2010 and 2012 were composed of two groups 27. Given that the majority of physical activity interventions, like most behavioural trials, are composed of multiple components, continuing to utilize the traditional two‐arm RCT restricts opportunity to advance effectiveness and secondly understand effects across various settings.

The identification of the key active ingredients in interventions is crucial towards generating knowledge that will enable the optimization and development of more effective interventions. Commonly, researchers perform exploratory analyses posthoc to understand differences in intervention components and settings. However, these tests are subject to confounding. Additionally, systematic reviews and accompanying meta‐regressions are regularly used to understand mediators and moderators of intervention effectiveness. Yet, the use of meta‐regressions to make inferences about individual level change, using study level information are, and will continue to be, at risk of ecological fallacy 28. Full and fractional factorial design trials and multi‐arm multi‐stage trials are methods of evaluation that enable multiple intervention components to be assessed simultaneously 29, 30. This includes isolation and testing both of characteristics of the intervention program, and aspects of implementation and delivery. These methods enable researchers to test intervention component hypotheses without the need for a full confirmatory trial until there is sufficient indication of effectiveness. Evaluating frameworks, including the multi‐phase optimization strategy, have been developed and tested for optimization 31. While multi‐arm and factorial methods have high short‐term resource requirements, we suggest that they may have the large long‐term savings in comparison to conducting sequential large‐scale trials. Given the influence of research funding requirements on researcher actions, it is crucial that funders recognize the long‐term benefits of investment in alternative research methods and support a shift in research towards newer methods of evaluation that require longer planning, implementation and evaluation times. Alongside, we highlight the use of individual level meta‐analyses and regressions as a non‐biased alternative to traditional meta‐analyses. While requiring significant researcher cooperation, the output is promising as demonstrated by InterConnect, a recently developed global database enabling federated meta‐analyses of the determinants of diabetes and obesity 32.

In light of the sufficiency and stability of the large evidence base of RCTs that has amassed since 1987, the results of this review raise a collective need for a new approach to intervention development and optimization focused on the scaling up and population impact of interventions. While the considerably more recent achievement of thresholds for longer term outcomes is promising, suggesting that over time we have moved towards a focus on longer term outcomes, the overall results direct a need to shift focus. The opportunity costs of continuing with the current repeated generation of two‐arm RCTs on the effects of interventions in highly controlled settings, without systematic consideration of longer term implementation within broader systems, is large for participants, researchers, funders and health systems. As a field, if we want to have a meaningful impact at a population scale, we need to work to fill the knowledge gap for the long‐term sustainability and effectiveness of evidence‐based practice and research 33. This includes addressing challenges in the implementation and embedment of programs within societal and governmental systems.

The two‐stage approach of this review has inherent weaknesses. Reliance on previous systematic reviews for the pool of primary data is subject to bias. In addition, only a proportion of studies were double coded and, therefore, we cannot rule out some inaccuracies due to coding. While we recognize that the literature searches were conducted in spring 2015, the inclusion of additional studies through up‐to‐date searches would only serve to further confirm the findings of our analyses. The measures of sufficiency and stability reported for both short and longer term effects suggest that multiple large trials with a drastically negative effect would be needed to invalidate our findings.

We acknowledge the multiple methodological options available for the evaluation of cumulative meta‐analyses. Given the lack of consensus on which of these are ‘best’, we selected the option we felt was most appropriate for this public health question. In post hoc analyses, we applied an alternative method which produced similar findings and would lead to the same conclusions as are currently drawn 34. Lastly, the nature of cumulative meta‐analysis as a sequential procedure in which an updated meta‐analysis is performed each time a new trial is added to the analysis, brings with it issues and risks with respect to repeated testing and inflated type one error 35.

In examining the accumulation of evidence, this paper is not suggesting that the 23 RCTs published after the thresholds of sufficiency and stability were achieved were not worth conducting. We recognize that the evidence generated from additional RCTs may resolve uncertainties for specific settings, mechanisms of intervention delivery and effectiveness. Rather, this paper suggests that a research field often establishes answers to research questions sooner than collectively realized and re‐emphasizes the importance of reflecting on the accumulated evidence base before proceeding with the generation of new evidence. In the face of the global inactivity pandemic, these results suggest that researchers must shift focus towards the development and optimization of interventions that can be effectively scaled‐up to achieve long‐term behaviour change across populations.

## Conflict of interest statement

No conflict of interest was declared.

## Supporting information

Data S1: Database Search Strategies
Data S2: Included primary studies and systematic reviews
Data S3: Excluded studies and reasons for exclusion
Data S4: Post intervention Cumulative Meta‐analysis
Data S5: Long term follow‐up Cumulative Meta‐analysis
Table 1: Reasons for exclusion of primary studies
Table 2: Application of indicators of sufficiency and stability
Table 3: Application of indicators of sufficiency and stability
Figure 1. Application of indicator or of sufficiency (Failsafe Ratio)
Figure 2. Application of indicator of stability (Cumulative Slope)
Figure 3. Application of indicator of sufficiency (Failsafe Ratio)
Figure 4. Application of indicator of stability (Cumulative Slope)
