# Supplementary material for: A cumulative meta‐analysis of the effects of individual physical activity interventions targeting healthy adults
Source: Obes Rev. 2018 Apr 27;19(8):1164–72. doi: 10.1111/obr.12690 (PMC6099338; doi:10.1111/obr.12690)

**Supplementary Files**

Contents

[S1: Database Search Strategies 1](#_Toc495347328)

[CENTRAL: 1](#_Toc495347329)

[MEDLINE: 2](#_Toc495347330)

[EMBASE Ovid: 3](#_Toc495347331)

[CINAHL Plus with Full Text EBSCO: 4](#_Toc495347332)

[Psyc Info: 5](#_Toc495347333)

[Web of Science: 6](#_Toc495347334)

[S2: Included primary studies and systematic reviews 6](#_Toc495347335)

[Reference list of included primary studies: 6](#_Toc495347336)

[Reference list of included systematic reviews: 12](#_Toc495347337)

[S3: Excluded studies and reasons for exclusion 17](#_Toc495347338)

[Reference list of excluded primary studies: 17](#_Toc495347339)

[Table 1: Reasons for exclusion of primary studies 28](#_Toc495347340)

[S4: Post intervention Cumulative Meta-analysis: 34](#_Toc495347341)

[Table 2: Application of indicators of sufficiency and stability 34](#_Toc495347342)

[Figure 1. Application of indicator or of sufficiency (Failsafe Ratio) 36](#_Toc495347343)

[Figure 2. Application of indicator of stability (Cumulative Slope) 37](#_Toc495347344)

[S5: Long term follow-up Cumulative Meta-analysis 38](#_Toc495347345)

[Table 3: Application of indicators of sufficiency and stability 38](#_Toc495347346)

[Figure 3. Application of indicator of sufficiency (Failsafe Ratio) 40](#_Toc495347347)

[Figure 4. Application of indicator of stability (Cumulative Slope) 41](#_Toc495347348)

## S1: Database Search Strategies

### CENTRAL:

#1MeSH descriptor: [Physical Fitness] this term only

#2MeSH descriptor: [Physical Exertion] this term only

#3MeSH descriptor: [Physical Education and Training] explode all trees

#4MeSH descriptor: [Sports] explode all trees

#5MeSH descriptor: [Dancing] this term only

#6MeSH descriptor: [Exercise Therapy] explode all trees

#7physical* near activ*

#8physical* near train*

#9physical* near fit*

#10exercise* near train*

#11exercise* near activ*

#12exercise* near physical*

#13sport*

#14walk*

#15bicycle*

#16exercise* near aerobic*

#17((life next style*) near activ*)

#18life-style* near activ*

#19lifestyle* near activ*

#20((life next style*) near physical*)

#21life-style* near physical*

#22lifestyle* near physical*

#23#1 or #2 or #3 or #4 or #5 or #6 or #7 or #8 or #9 or #10 or #11

#24#12 or #13 or #14 or #15 or #16 or #17 or #18 or #19 or #20 or #21 or #22

#25#23 or #24

#26MeSH descriptor: [Health Education] this term only

#27MeSH descriptor: [Patient Education as Topic] this term only

#28MeSH descriptor: [Primary Prevention] this term only

#29MeSH descriptor: [Health Promotion] explode all trees

#30MeSH descriptor: [Behavior Therapy] this term only

#31MeSH descriptor: [Cognitive Therapy] this term only

#32MeSH descriptor: [Primary Health Care] this term only

#33MeSH descriptor: [Workplace] this term only

#34promot*

#35educat*

#36program*

#37#26 or #27 or #28 or #29 or #30 or #31 or #32 or #33 or #34 or #35 or #36

#38#25 and #37

### MEDLINE:

1. Physical Exertion/

2. Physical Fitness/

3. exp “Physical Education and Training”/

4. exp Sports/

5. Dancing/

6. exp Exercise therapy/

7. exp Exercise/

8. (physical$ adj5 (fit$ or train$ or activ$ or endur$ or exertion$)).tw.

9. (exercis$ adj5 (train$ or physical$ or activ$)).tw.

10. sport$.tw.

11. walk$.tw.

12. bicycle$.tw.

13. ((exercise$ adj3 aerobic$) or aerobics).tw.

14. ((lifestyle or life-style) adj5 activ$).tw.

15. ((lifestyle or life-style) adj5 physical$).tw.

16. or/1-15

17. Health Education/

18. Patient Education as Topic/

19. Primary Prevention/

20. exp Health Promotion/

21. Behavior Therapy/

22. Cognitive Therapy/

23. Primary Health Care/

24.Workplace/

25. promot$.tw.

26. educat$.tw.

27. program$.tw.

28. or/17-27

29. 16 and 28

30. Meta-Analysis as Topic/

31. meta analy$.tw.

32. metaanaly$.tw.

33. Meta-Analysis/

34. (systematic adj (review$1 or overview$1)).tw.

35. exp Review Literature as Topic/

36. or/30-35

37. cochrane.ab.

38. embase.ab.

39. (psychlit or psyclit).ab.

40. (cinahl or cinhal).ab.

41. science citation index.ab.

42. bids.ab.

43. cancerlit.ab.

44. or/37-43

45. reference list$.ab.

46. bibliograph$.ab.

47. hand-search$.ab.

48. relevant journals.ab.

49. manual search$.ab.

50. or/45-49

51. selection criteria.ab.

52. data extraction.ab.

53. 51 or 52

54. Review/

55. 53 and 54

56. Comment/

57. Letter/

58. Editorial/

59. animal/

60. human/

61. 59 not (59 and 60)

62. or/56-58,61

63. 36 or 44 or 50 or 53

64. 63 not 62

### EMBASE Ovid:

1. exp exercise/

2. fitness/

3. physical education/

4. exp sport/

5. dancing/

6. exp kinesiotherapy/

7. (physical* adj5 (fit* or train* or activ* or endur* or exert*)).tw.

8. (exercis* adj5 (train* or physical* or activ*)).tw.

9. sport*.tw.

10. walk*.tw.

11. ((exercise* adj aerobic*) or aerobic*).tw.

12. ((lifestyle or life-style) adj5 activ*).tw.

13. bicycle*.tw.

14. ((lifestyle or life-style) adj5 physical*).tw.

15. or/1-14

16. health education/

17. patient education/

18. primary prevention/

19. health promotion/

20. behavior therapy/

21. cognitive therapy/

22. primary health care/

23. workplace/

24. promot*.tw.

25. educat*.tw.

26. program*.tw.

27. or/16-26

28. 15 and 27

29. exp Meta Analysis/

30. ((meta adj analy$) or metaanalys$).tw.

31. (systematic adj (review$1 or overview$1)).tw.

32. or/29-31

33. cancerlit.ab.

34. cochrane.ab.

35. embase.ab.

36. (psychlit or psyclit).ab.

37. (psychinfo or psycinfo).ab.

38. (cinahl or cinhal).ab.

39. science citation index.ab.

40. bids.ab.

41. or/33-40

42. reference lists.ab.

43. bibliograph$.ab.

44. hand-search$.ab.

45. manual search$.ab.

46. relevant journals.ab.

47. or/42-46

48. data extraction.ab.

49. selection criteria.ab.

50. 48 or 49

51. review.pt.

52. 50 and 51

53. letter.pt.

54. editorial.pt.

55. animal/

56. human/

57. 55 not (55 and 56)

58. or 53-54,57

59. 32 or 41 or 47 or 52

### CINAHL Plus with Full Text EBSCO:

S21 + systematic review filter

S20 S10 and S19

S19 S11 or S12 or S13 or S14 or S15 or S16 or S17 or S18

S18 (TI promot* or educat* or program*) or (AB promot* or educat* or program*)

S17 (MH “Work Environment”)

S16 (MH “Primary Health Care”)

S15 (MH “Behavior Therapy+”)

S14 (MH “Health Promotion”)

S13 (MH “Preventive Health Care”)

S12 (MH “Patient Education”)

S11 (MH “Health Education”)

S10 S1 or S2 or S3 or S4 or S5 or S6 or S7 or S8 or S9

S9 (TI sport* or walk* or bicycle* or exercis* or aerobic*) or (AB sport* or walk* or bicycle* or exercis* or aerobic*)

S8 (TI physical N5 (fit* or train* or activ* or endur* or exert*)) or (AB physcial* N5 (fit* or train* or activ* or endur* or exert*))

S7 (TI exercis* N5 (train* or physical* or activ*)) or (AB exercis* N5 (train* or physical* or activ*))

S6 (MH “Exercise+”) or (MH “Therapeutic Exercise+”)

S5 (TI (lifestyle* or life-style*)N5 (activ* or physical*)) or (AB (lifestyle* or life-style*)N5 (activ* or physical*))

S4 (MH “Sports+”) or (MH “Dancing+”)

S3 (MH “Physical Education and Training”)

S2 (MH “Physical Fitness”)

S1 (MH “Exertion”)

### Psyc Info:

1. exp exercise/

2. physical fitness/

3. physical activity/

4. exp sports/

5. physical education

6. (physical$ adj5 (fit$ or train$ or activ$ or endur$ or exertion$)).tw.

7. (exercis$ adj5 (train$ or physical$ or activ$)).tw.

8. sport$.tw.

9. walk$.tw.

10. bicycle$.tw.

11. ((exercise$ adj3 aerobic$) or aerobics).tw.

12. ((lifestyle or life-style) adj5 activ$).tw.

13. ((lifestyle or life-style) adj5 physical$).tw.

14. or/1-13

15. health education/

16. client education/

17. health promotion/

18. prevention/

19. primary health care/

20. behavior therapy/

21. cognitive therapy/

22. cognitive behavior therapy/

23. workplace*.tw.

24. promot$.tw.

25. educat$.tw.

26. program$.tw.

27. or/15-26

28. 14 and 27

29. + systematic review filter

### Web of Science:

# 20 #19 AND #18

# 19 + systematic review filter

# 18 #17 AND #8

# 17 #16 OR #15 OR #14 OR #13 OR #12 OR #11 OR #10 OR #9

# 16 TI=(promot* or educat* or program*)

# 15 TS=(workplace)

# 14 TS=(primary health care)

# 13 TS=(cognitive therap*)

# 12 TS=((behaviour or behavior) NEAR/2 therap*)

# 11 TS=(health NEAR/2 promot*)

# 10 TS=(primary prevent*)

# 9 TS=((health educat*) or (patient* educat*))

# 8 #7 OR #6 OR #5 OR #4 OR #3 OR #2 OR #1

# 7 TS=((lifestyle* or life-style*)NEAR/5 (activ* or physcial*))

# 6 TS=((exercis* NEAR/2 aerobic*) or aerobic*)

# 5 TS=(sport* or danc* or walk* or bicycle*)

# 4 TS=(physical* educat*)

# 3 TS=(exercis* NEAR/5 (train* or physical* or activ*))

# 2 TS=(physical NEAR/5 (fit* or train* or activ* or endur* or exert*))

# 1 TS=(exercis* therap*)

##

## S2: Included primary studies and systematic reviews

### Reference list of included primary studies:

**Aittasalo et al. 2004**

Aittasalo, M., Miilunpalo, S., & Suni, J. (2004). The effectiveness of physical activity counseling

in a work-site setting: A randomized, controlled trial. *Patient Education and Counseling*, *55*(2),

193–202.

**Baker et al. 2008**

Baker, G., Gray, S. R., Wright, A., Fitzsimons, C., Nimmo, M., Lowry, R., & Mutrie, N. (2008).

The effect of a pedometer-based community walking intervention “Walking for Wellbeing in the

West” on physical activity levels and health outcomes: A 12-week randomized controlled trial. *The*

*International Journal of Behavioral Nutrition and Physical Activity*.

**Bennett et al. 2008**

Bennett, J. a, Young, H. M., Nail, L. M., Winters-Stone, K., & Hanson, G. (2008). A telephoneonly

motivational intervention to increase physical activity in rural adults: a randomized controlled

trial. *Nursing Research*, *57*(1), 24–32.

**Blissmer & McAuley 2002**

Blissmer, B., & McAuley, E. (2002). Testing the requirements of stages of physical activity among

adults: the comparative effectiveness of stage-matched, mismatched, standard care, and control

interventions. *Annals of al Medicine!: A Publication of the Society of Behavioral Medicine*, *24*(3),

181–189.

**Buman et al. 2011**

Buman, M. P., Giacobbi, P. R., Dzierzewski, J. M., Aiken Morgan, A., McCrae, C. S., Roberts, B.

L., & Marsiske, M. (2011). Peer volunteers improve long-term maintenance of physical activity

with older adults: a randomized controlled trial. *Journal of Physical Activity & Health*, *8* (Suppl 2),

S257–66.

**Campbell et al. 2002**

Campbell, M. K., Tessaro, I., DeVellis, B., Benedict, S., Kelsey, K., Belton, L., & Sanhueza, A.

(2002). Effects of a tailored health promotion program for female blue-collar workers: health works

for women. *Preventive Medicine*, *34*(3), 313–323.

**Campbell et al. 2004**

Campbell, M. K., James, A., Hudson, M. a, Carr, C., Jackson, E., Oakes, V., … Tessaro, I. (2004).

Improving multiple behaviors for colorectal cancer prevention among african american church

members. *Health Psychology!: Official Journal of the Division of Health Psychology, American*

*Psychological Association*, *23*(5), 492–502.

**Castro et al. 2011**

Castro, C. M., Pruitt, L. a, Buman, M. P., & King, A. C. (2011). Physical activity program delivery

by professionals versus volunteers: the TEAM randomized trial. *Health Psychology!: Official*

*Journal of the Division of Health Psychology, American Psychological Association*, *30*(3), 285–

294.

**Chang et al. 2010**

Chang, M. W., Nitzke, S., & Brown, R. (2010). Design and Outcomes of a Mothers In Motion

Behavioral Intervention Pilot Study. *Journal of Nutrition Education and Behavior*, *42*(3 SUPPL.),

S11–S21.

**Cook et al. 2001**

Cook, C., Simmons, G., Swinburn, B., & Stewart, J. (2001). Changing risk behaviours for noncommunicable disease in New Zealand working men--is workplace intervention effective? *The*

*New Zealand Medical Journal*, *114*(1130), 175–178.

**Cook et al. 2007**

Cook, R., Billings, D., Hersch, R., Back, A., Hendrickson, A. (2007). A Field Test of a Web-Based

Workplace Health Promotion Program to Improve Dietary Practices, Reduce Stress, and Increase

Physical Activity: Randomized Controlled Trial. *Journal of Medical Internet Research,* 9(2): e17.

**Cunningham et al. 1987**

Cunningham, D. a, Rechnitzer, P. a, Howard, J. H., & Donner, a P. (1987). Exercise training of

men at retirement: a clinical trial. *Journal of Gerontology*, *42*(1), 17–23.

**Cussler et al. 2008**

Cussler, E. C., Teixeira, P. J., Going, S. B., Houtkooper, L. B., Metcalfe, L. L., Blew, R. M., …

Lohman, T. G. (2008). Maintenance of weight loss in overweight middle-aged women through the

Internet. *Obesity (Silver Spring, Md.)*, *16*(5), 1052–1060.

**De Jong et al. 2007**

De Jong, J., Lemmink, K. a P. M., King, A. C., Huisman, M., & Stevens, M. (2007). Twelve-month

effects of the Groningen active living model (GALM) on physical activity, health and fitness

outcomes in sedentary and underactive older adults aged 55-65. *Patient Education and Counseling*,

*66*(2), 167–176.

**Dishman et al. 2009**

Dishman, R. K., DeJoy, D. M., Wilson, M. G., & Vandenberg, R. J. (2009). Move to Improve. A

Randomized Workplace Trial to Increase Physical Activity. *American Journal of Preventive*

*Medicine*, *36*(2), 133–141.

**Dirige et al. 2013**

Dirige, O. V., Carlson, J. a., Alcaraz, J., Moy, K. L., Rock, C. L., Oades, R., & Sallis, J. F. (2013).

Siglang Buhay: Nutrition and Physical Activity Promotion in Filipino-Americans Through

Community Organizations. *Journal of Public Health Management and Practice*, *19*(2), 162–168.

**Dunton and Robertson. 2008**

Dunton, G. F., & Robertson, T. P. (2008). A tailored internet-plus-email intervention for increasing

physical activity among ethnically-diverse women. *Preventive Medicine*, *47*(6), 605–611.

**Eiben & Lissner 2006**

Eiben, G., & Lissner, L. (2006). Health Hunters--an intervention to prevent overweight and obesity

in young high-risk women. *International Journal of Obesity (2005)*, *30*(4), 691–696.

**Elley et al. 2003**

Elley, R., Kerse, N., Arroll, B., Robinson, E. (2003). Effectiveness of counselling patients on

physical activity in general practice: a cluster randomised controlled trial. *The Journal of the Royal*

*College of General Practitioners*, *29*(205), 502.

**Fahrenwald et al. 2004**

Fahrenwald, N. L., Atwood, J. R., Walker, S. N., Johnson, D. R., & Berg, K. (2004). A randomized

pilot test of “Moms on the Move”: a physical activity intervention for WIC mothers. *Annals of*

*Behavioral Medicine!: A Publication of the Society of Behavioral Medicine*, *27*(2), 82–90.

**Fjeldsoe et al. 2010**

Fjeldsoe, B. S., Miller, Y. D., & Marshall, A. L. (2010). MobileMums: A randomized controlled

trial of an SMS-based physical activity intervention. *Annals of Behavioral Medicine*, *39*(2), 101–

111.

**Goldstein et al. 1999**

Goldstein, M. G., Pinto, B. M., Ph, D., Marcus, B. H., Lynn, H., Jette, A. M., … Tennstedt, S.

(1999). Physician-based physical activity counseling for middle-aged and older adults!: a

randomized trial. *Annals of Behavioral Medicine*, *21*(1), 40–47.

**Grandes et al. 2011**

Grandes, G., Sanchez, A., Montoya, I., Ortega Sanchez-Pinilla, R., & Torcal, J. (2011). Two-year

longitudinal analysis of a cluster randomized trial of physical activity promotion by general

practitioners. *PloS One*, *6*(3), e18363.

**Green et al. 2002**

Green, B. B., McAfee, T., Hindmarsh, M., Madsen, L., Caplow, M., & Buist, D. (2002).

Effectiveness of telephone support in increasing physical activity levels in primary care patients.

*American Journal of Preventive Medicine*, *22*(3), 177–183.

**Hager et al. 2002**

Hager, R. L., Hardy, A., Aldana, S. G., & George, J. D. (2002). Evaluation of an Internet, Stage-

Based Physical Activity Intervention. *American Journal of Health Education*, *33*(6), 329–337.

**Hillsdon et al. 2002**

Hillsdon, M., Thorogood, M., White, I., & Foster, C. (2002). Advising people to take more exercise

is ineffective: a randomized controlled trial of physical activity promotion in primary care.

*International Journal of Epidemiology*, *31*(4), 808–815.

**Hurling et al. 2007**

Hurling, R., Catt, M., DeBoni, M., Fairley, B., Hurst, T., Murrary, P., Richardson, A., Sodhi, J.

(2007). Using Internet and Mobile Phone Technology to Deliver an Automated Physical Activity

Program: Randomized Controlled Trial. *Journal of Medical Internet Research,* 9(2): e7

**Katz et al. 2008**

Katz, D. L., Shuval, K., Comerford, B. P., Faridi, Z., & Njike, V. Y. (2008). Impact of an

educational intervention on internal medicine residents’ physical activity counselling: The Pressure

System Model. *Journal of Evaluation in Clinical Practice*, *14*(2), 294–299.

**Keyserling et al. 2008**

Keyserling, T. C., Samuel Hodge, C. D., Jilcott, S. B., Johnston, L. F., Garcia, B. a., Gizlice, Z., …

Ammerman, A. S. (2008). Randomized trial of a clinic-based, community-supported, lifestyle

intervention to improve physical activity and diet: The North Carolina enhanced WISEWOMAN

project. *Preventive Medicine*, *46*(6), 499–510.

**King et al. 1988**

King, a C., Taylor, C. B., Haskell, W. L., & Debusk, R. F. (1988). Strategies for increasing early

adherence to and long-term maintenance of home-based exercise training in healthy middle-aged

men and women. *The American Journal of Cardiology*, *61*(8), 628–632.

**King et al. 2007**

King, A. C., Friedman, R., Marcus, B., Castro, C., Napolitano, M., Ahn, D., & Baker, L. (2007).

Ongoing physical activity advice by humans versus computers: the Community Health Advice by

Telephone (CHAT) trial. *Health Psychology!: Official Journal of the Division of Health*

*Psychology, American Psychological Association*, *26*(6), 718–727.

**King et al. 2008**

King, A. C., Ahn, D. K., Oliveira, B. M., Atienza, A. a., Castro, C. M., & Gardner, C. D. (2008).

Promoting Physical Activity Through Hand-Held Computer Technology. *American Journal of*

*Preventive Medicine*, *34*(2), 138–142.

**Kinmonth et al. 2008**

Kinmonth, A. L., Wareham, N. J., Hardeman, W., Sutton, S., Prevost, a. T., Fanshawe, T., …

Griffin, S. J. (2008). Efficacy of a theory-based behavioural intervention to increase physical

activity in an at-risk group in primary care (ProActive UK): a randomised trial. *The Lancet*,

*371*(9606), 41–48.

**Kirkwood et al. 2007**

Kirkwood, L., Aldujaili, E., & Drummond, S. (2007). Effects of advice on dietary intake and/or

physical activity on body composition, blood lipids and insulin resistance following a low-fat,

sucrose-containing, high-carbohydrate, energy-restricted diet. *International Journal of Food*

*Sciences and Nutrition*, *58*(5), 383–397.

**Lee & White 1997**

Lee, C., & White, S. W. (1997). Controlled trial of a minimal-intervention exercise program for

middle-aged working women. *Psychology & Health*, *12*(3), 361–374.

**Loughlan & Mutrie 1997**

Loughlan, C., & Mutrie, N. (1997). An evaluation of the effectiveness of three interventions in

promoting physical activity in a sedentary population. *Health Education Journal*, *56*(2), 154–165.

**Lowther et al. 2002**

Lowther, M., Mutrie, N., & Scott, E. M. (2002). Promoting physical activity in a socially and

economically deprived community: a 12 month randomized control trial of fitness assessment and

exercise consultation. *Journal of Sports Sciences*, *20*(7), 577–588.

**Marcus et al. 2007**

Marcus, B. H., Napolitano, M. a, King, A. C., Lewis, B. a, Whiteley, J. a, Albrecht, A., …

Papandonatos, G. D. (2007). Telephone versus print delivery of an individualized motivationally

tailored physical activity intervention: Project STRIDE. *Health Psychology!: Official Journal of the*

*Division of Health Psychology, American Psychological Association*, *26*(4), 401–409.

**Merom et al. 2007**

Merom, D., Rissel, C., Phongsavan, P., Smith, B. J., Van Kemenade, C., Brown, W. J., & Bauman,

A. E. (2007). Promoting Walking with Pedometers in the Community. The Step-by-Step Trial.

*American Journal of Preventive Medicine*, *32*(4), 290–297.

**Morgan et al. 2009**

Morgan, P. J., Lubans, D. R., Collins, C. E., Warren, J. M., & Callister, R. (2009). The SHED-IT

randomized controlled trial: evaluation of an Internet-based weight-loss program for men. *Obesity*

*(Silver Spring, Md.)*, *17*(11), 2025–2032.

**Napolitano et al. 2003**

Napolitano, M. a, Fotheringham, M., Tate, D., Sciamanna, C., Leslie, E., Owen, N., … Marcus, B.

(2003). Evaluation of an internet-based physical activity intervention: a preliminary investigation.

*Annals of Behavioral Medicine!: A Publication of the Society of Behavioral Medicine*, *25*(2), 92–

99.

**Napolitano et al. 2006**

Napolitano, M. a., Whiteley, J. a., Papandonatos, G., Dutton, G., Farrell, N. C., Albrecht, A., …

Marcus, B. H. (2006). Outcomes from the women’s wellness project: A community-focused

physical activity trial for women. *Preventive Medicine*, *43*(6), 447–453.

**Newton & Perri. 2004**

Newton, R. L., & Perri, M. G. (2004). A randomized pilot trial of exercise promotion in sedentary

African-American adults. *Ethnicity and Disease*, *14*(4), 548–557.

**Openacker et al. 2008**

Opdenacker, J., Boen, F., Vanden Auweele, Y., & De Bourdeaudhuij, I. (2008). Effectiveness of a

lifestyle physical activity intervention in a women’s organization. *Journal of Women’s Health*

*(2002)*, *17*(3), 413–421.

**Peterson et al. 2005**

Peterson, J. A., Yates, B. C., Atwood, J. R., & Hertzog, M. (2005). Effects of a physical activity

intervention for women. *Western Journal of Nursing Research*, *27*(1), 93–110.

**Pinto et al. 2002**

Pinto, B. M., Friedman, R., Marcus, B. H., Kelley, H., Tennstedt, S., & Gillman, M. W. (2002).

Effects of a Computer-Based, Telephone-Counseling System on Physical Activity. *American*

*Journal of Preventive Medicine*, *23*(2), 113–120.

**Proper et al. 2003**

Proper, K. I., Hildebrandt, V. H., Van Der Beek, A. J., Twisk, J. W. R., & Van Mechelen, W.

(2003). Effect of individual counseling on physical activity fitness and health: A randomized

controlled trial in a workplace setting. *American Journal of Preventive Medicine*, *24*(3), 218–226.

**Resnicow et al. 2005**

Resnicow, K., Jackson, A., Blissett, D., Wang, T., McCarty, F., Rahotep, S., & Periasamy, S.

(2005). Results of the healthy body healthy spirit trial. *Health Psychology!: Official Journal of the*

*Division of Health Psychology, American Psychological Association*, *24*(4), 339–348.

**Simons-Morton et al. 2001**

Simons-Morton, D., Blair, S., King, A., Morgan, W., Applegate, M., et al. (2001). Effects of

physical activity counseling in primary care: the Activity Counseling Trial: a randomized

controlled trial. *Journal of the American Medical Association.* 286(6):677–87.

**Smith et al. 2000**

Smith, B. J., Bauman, a E., Bull, F. C., Booth, M. L., & Harris, M. F. (2000). Promoting physical

activity in general practice: a controlled trial of written advice and information materials. *British*

*Journal of Sports Medicine*, *34*(4), 262–267.

**Spittaels et al. 2007**

Spittaels, H., De Bourdeaudhuij, I., Brug, J., & Vandelanotte, C. (2007). Effectiveness of an online

computer-tailored physical activity intervention in a real-life setting. *Health Education Research*,

*22*(3), 385–396.

**Staten et al. 2004**

Staten, L. K., Gregory-Mercado, K. Y., Ranger-Moore, J., Will, J. C., Giuliano, A. R., Ford, E. S.,

& Marshall, J. (2004). Provider counseling, health education, and community health workers: the

Arizona WISEWOMAN project. *Journal of Women’s Health (2002)*, *13*(5), 547–556.

**Stevens et al. 1998**

Stevens, W., Hillsdon, M., Thorogood, M., & McArdle, D. (1998). Cost-effectiveness of a primary

care based physical activity intervention in 45-74 year old men and women: a randomised

controlled trial. *British Journal of Sports Medicine*, *32*(3), 236–241.

**Thompson et al. 2008**

Thompson, J. L., Allen, P., Helitzer, D. L., Qualls, C., Whyte, A. N., Wolfe, V. K., & Herman, C.

J. (2008). Reducing Diabetes Risk in American Indian Women. *American Journal of Preventive*

*Medicine*, *34*(3), 192–201.

**Van Keulen 2011**

Van Keulen, H. M., Mesters, I., Ausems, M., Van Breukelen, G., Campbell, M., Resnicow, K., …

De Vries, H. (2011). Tailored print communication and telephone motivational interviewing are

equally successful in improving multiple lifestyle behaviors in a randomized controlled trial.

*Annals of Behavioral Medicine*, *41*(1), 104–118.

**Van Stralen et al. 2011**

Van Stralen, M. M., de Vries, H., Mudde, A. N., Bolman, C., & Lechner, L. (2011). The long-term

efficacy of two computer-tailored physical activity interventions for older adults: main effects and

mediators. *Health Psychology!: Official Journal of the Division of Health Psychology, American*

*Psychological Association*, *30*(4), 442–452.

**Van Wier et al. 2009**

Van Wier, M. F., Ariëns, G. a M., Dekkers, J. C., Hendriksen, I. J. M., Smid, T., & van Mechelen,

W. (2009). Phone and e-mail counselling are effective for weight management in an overweight

working population: a randomized controlled trial. *BMC Public Health*, *9*, 6.

**Winett et al. 2007**

Winett, R. a., Anderson, E. S., Wojcik, J. R., Winett, S. G., & Bowden, T. (2007). Guide to health:

Nutrition and physical activity outcomes of a group-randomized trial of an internet-based

intervention in churches. *Annals of Behavioral Medicine*, *33*(3), 251–261.

**Young & Stewart 2006**

Young, D. R., & Stewart, K. J. (2006). A church-based physical activity intervention for African

American women. *Family & Community Health*, *29*(2), 103–17.

### Reference list of included systematic reviews:

**Amiri Farahani et al. 2015**

Amiri Farahani, L., Asadi-Lari, M., Mohammadi, E., Parvizy, S., Haghdoost, a. a., & Taghizadeh, Z.

(2015). Community-based physical activity interventions among women: a systematic review. *BMJ*

*Open*, *5*(4), e007210–e007210.

**Barr-Anderson et al. 2011**

Barr-Anderson, D. J., Auyoung, M., Whitt-Glover, M. C., Glenn, B. a., & Yancey, A. K. (2011).

Integration of short bouts of physical activity into organizational routine: A systematic review of the

literature. *American Journal of Preventive Medicine*, *40*(1), 76–93.

**Bender et al. 2014**

Bender, M. S., Choi, J., Won, G. Y., & Fukuoka, Y. (2014). Randomized controlled trial lifestyle

interventions for Asian Americans: A systematic review. *Preventive Medicine*, *67*, 171–181.

**Bock et al. 2014**

Bock, C., Jarczok, M. N., & Litaker, D. (2014). Community-based efforts to promote physical

activity: a systematic review of interventions considering mode of delivery, study quality and

population subgroups. *Journal of Science and Medicine in Sport / Sports Medicine Australia*, *17*(3),

276–282.

**Bravata et al. 2007**

Bravata, Dena M, Smith-Spangler, Crystal, Sundaram, Vandana, Giender, Alison, Lin, Nancy,

Lewis, Robyn, Stave, Christopher, Olkin, Ingram, Sirard, J. (2007). Using Pedometers to Increase

Physical Activity: A Systematic Review, *298*(19).

**Broekhuizen et al. 2012**

Broekhuizen, K., Kroeze, W., van Poppel, M. N. M., Oenema, A., & Brug, J. (2012). A systematic

review of randomized controlled trials on the effectiveness of computer-tailored physical activity

and dietary behavior promotion programs: An update. *Annals of Behavioral Medicine*.

**Buchholz et al. 2013**

Buchholz, S. W., Wilbur, J., Ingram, D., & Fogg, L. (2013). Physical activity text messaging

interventions in adults: A systematic review. *Worldviews on Evidence-Based Nursing*.

**Buhi et al. 2012**

Buhi, E. R., Trudnak, T. E., Martinasek, M. P., Oberne, A. B., Fuhrmann, H. J., & McDermott, R. J.

(2012). Mobile phone-based behavioural interventions for health: A systematic review. *Health*

*Education Journal*.

**Carroll et al. 2008**

Carroll, J. K., Fiscella, K., Epstein, R. M., Jean-Pierre, P., Figueroa-Moseley, C., Williams, G. C.,

… Morrow, G. R. (2008). Getting patients to exercise more: A systematic review of underserved

populations. *The Journal of Family Practice*.

**Cavill et al. 2012**

Cavill, J.-L., Jancey, J. M., & Howat, P. (2012). Review and recommendations for online physical

activity and nutrition programmes targeted at over 40s. *Global Health Promotion*, *19*(2), 44–53.

**Chapman et al. 2013**

Chapman, J., Qureshi, N., & Kai, J. (2013). Effectiveness of physical activity and dietary

interventions in South Asian populations: a systematic review. *The British Journal of General*

*Practice!: The Journal of the Royal College of General Practitioners*, *63*(607), e104–14.

**Chau et al. 2010**

Chau, J. Y., van der Ploeg, H. P., van Uffelen, J. G. Z., Wong, J., Riphagen, I., Healy, G. N., …

Brown, W. J. (2010). Are workplace interventions to reduce sitting effective? A systematic review.

*Preventive Medicine: An International Journal Devoted to Practice and Theory*.

**Cleland et al. 2012**

Cleland, C. L., Tully, M. A., Kee, F., & Cupples, M. E. (2012). The effectiveness of physical

activity interventions in socio-economically disadvantaged communities: A systematic review.

*Preventive Medicine: An International Journal Devoted to Practice and Theory*.

**Cleland et al. 2013**

Cleland, V., Granados, a., Crawford, D., Winzenberg, T., & Ball, K. (2013). Effectiveness of

interventions to promote physical activity among socioeconomically disadvantaged women: A

systematic review and meta-analysis. *Obesity Reviews*, *14*(3), 197–212.

**Davies et al. 2012**

Davies, C. A., Spence, J. C., Vandelanotte, C., Caperchione, C. M., & Mummery, W. K. (2012).

Meta-analysis of internet-delivered interventions to increase physical activity levels. *The*

*International Journal of Behavioral Nutrition and Physical Activity*.

**Denison et al. 2014**

Denison, E., Vist, G. E., Underland, V., & Berg, R. C. (2014). Interventions aimed at increasing the

level of physical activity by including organised follow-up: a systematic review of effect. *BMC*

*Family Practice*, *15*, 120.

**Dishman et al. 1998**

Dishman, R. K., Oldenburg, B., O’Neal, H., & Shephard, R. J. (1998). Worksite physical activity

interventions. *American Journal of Preventive Medicine*. Netherlands: Elsevier Science.

**Dugill 2007**

Dugdill. (2007). *A Review of Effectiveness of Workplace Health Promotion Interventions on*

*Physical Activity and What Works in Motivating and Changing Employees’ Health Behaviour*.

**Eaton & Menard. 1998**

Eaton, C. B., & Menard, L. M. (1998). A systematic review of physical activity promotion in

primary care office settings. *British Journal of Sports Medicine*, *32*(1), 11–16.

**Eden et al. 2002**

Eden, Orleans, C. T., Mulrow, C. D., Pender, N. J., & Teutsch, S. M. (2002). Clinical Guidelines

Does Counseling by Clinicians Improve Physical Activity? A Summary for the U.S. Preventive

Task Force. *Annals of Internal Medicine*, *137*(3), 208–215.

**Foster et al. 2013**

Foster, C., Richards, J., Thorogood, M., & Hillsdon, M. (2013). Remote and web 2 . 0 interventions

for promoting physical activity ( Review ). *The Cochrane Database of Systematic Reviews*, (9).

**Freak-Poli et al. 2013**

Freak-Poli, R. L. A., Cumpston, M., Peeters, A., Clemes, S. A., Freak-Poli Rosanne, L. A.,

Cumpston, M., … Clemes Stacy, A. (2013). Workplace pedometer interventions for increasing

physical activity. *Cochrane Database of Systematic Reviews*, *4*(4), CD009209.

**Gourlan et al. 2011**

Gourlan, M. J., Trouilloud, D. O., & Sarrazin, P. G. (2011). Interventions promoting physical

activity among obese populations: A meta-analysis considering global effect, long-term

maintenance, physical activity indicators and dose characteristics. *Obesity Reviews*, *12*(7), 633–645.

**Hillsdon et al. 2005**

Hillsdon, M., Foster, C., & Thorogood, M. (2005). Interventions for promoting physical activity.

*The Cochrane Database of Systematic Reviews*, (1), CD003180.

**Hillsdon & Thorogood. 1996**

Hillsdon, M., & Thorogood, M. (1996). A systematic review of physical activity promotion

strategies. *British Journal of Sports Medicine*, *30*(2), 84–89.

**Hillsdon et al. 1995**

Hillsdon, M., Thorogood, M., Anstiss, T., & Morris, J. (1995). Randomised controlled trials of

physical activity promotion in free living populations: a review. *Journal of Epidemiology and*

*Community Health*, *49*(5), 448–453.

**Kahn et al. 2010**

Kahn, E., Ramsey, L., Brownson, R., Heath, G., Howze, E., Powell, K., … Rajab, M. (2010). The

Effectiveness of Interventions A Systematic Review. *American Journal of Preventive Medicine*,

*22*(02).

**Kassavou et al. 2013**

Kassavou, A., Turner, A., & French, D. P. (2013). Do interventions to promote walking in groups

increase physical activity? A meta-analysis. *The International Journal of Behavioral Nutrition and*

*Physical Activity*.

**Lemacks et al. 2013**

Lemacks, J., Wells, B. A., Ilich, J. Z., & Ralston, P. A. (2013). Interventions for improving nutrition

and physical activity behaviors in adult African American populations: a systematic review, January

2000 through December 2011. *Preventing Chronic Disease*, *10*, E99.

**Malik et al. 2014**

Malik, S. H., Blake, H., & Suggs, L. S. (2014). A systematic review of workplace health promotion

interventions for increasing physical activity. *British Journal of Health Psychology*.

**Martin-Ginis et al. 2013**

Martin-, Ginis, K. A. M., Nigg, C. R., Smith, A. L., Martin Ginis, K. A., Nigg, C. R., & Smith, A. L.

(2013). Peer-delivered physical activity interventions: An overlooked opportunity for physical

activity promotion. *Translational Behavioral Medicine*, *3*(4), 434–443.

**Morgan et al. 2005**

Morgan, O. (2005). Approaches to increase physical activity: reviewing the evidence for exercisereferral

schemes. *Public Health*, *119*(5), 361–370.

**Müller-Riemenschneider et al. 2008**

Müller-Riemenschneider, F., Reinhold, T., Nocon, M., & Willich, S. N. (2008). Long-term

effectiveness of interventions promoting physical activity: A systematic review. *Preventive*

*Medicine*, *47*(4), 354–368.

**Neville et al. 2009**

Neville, L. M., O’Hara, B., & Milat, A. (2009). Computer-tailored physical activity behavior change

interventions targeting adults: A systematic review. *The International Journal of Behavioral*

*Nutrition and Physical Activity*.

**Newton et al. 2014**

Newton, R. L., Griffith, D. M., Kearney, W. B., & Bennett, G. G. (2014). A systematic review of

weight loss, physical activity and dietary interventions involving African American men. *Obesity*

*Reviews*, *15*, 93–106.

**Olgivie et al. 2007**

Ogilvie, D., Foster, C. E., Rothnie, H., Cavill, N., Hamilton, V., Fitzsimons, C. F., & Mutrie, N.

(2007). Interventions to promote walking: systematic review. *BMJ (Clinical Research Ed.)*,

*334*(7605), 1204.

**Orrow et al. 2012**

Orrow, G., Kinmonth, A.-L., Sanderson, S., & Sutton, S. (2012). Effectiveness of physical activity

promotion based in primary care: systematic review and meta-analysis of randomised controlled

trials. *BMJ (Clinical Research Ed.)*, *344*, e1389.

**Perez et al. 2010**

Perez, A., Fleury, J., & Keller, C. (2010). Review of intervention studies promoting physical activity

in Hispanic women. *Western Journal of Nursing Research*. Sage Publications.

**Petrella & Lattanzio. 2002**

Petrella, R. J., & Lattanzio, C. N. (2002). Does counseling help patients get active? Systematic

review of the literature. *Canadian Family Physician Medecin de Famille Canadien*, *48*, 72–80.

**Priest et al. 2008**

Priest, N., Armstrong, R., Doyle, J., & Waters, E. (2008). Interventions implemented through

sporting organisations for increasing participation in sport. *The Cochrane Database of Systematic*

*Reviews*, (3), CD004812.

**Proper et al. 2003**

Proper, K. I., Koning, M., van der Beek, A. J., Hildebrandt, V. H., Bosscher, R. J., & van Mechelen,

W. (2003). The effectiveness of worksite physical activity programs on physical activity, physical

fitness, and health. *Clinical Journal of Sport Medicine!: Official Journal of the Canadian Academy*

*of Sport Medicine*, *13*(2), 106–117.

**Richards et al. 2013**

Richards, J., Hillsdon, M., Thorogood, M., & Foster, C. (2013). Face-to-face interventions for

promoting physical activity. *The Cochrane Database of Systematic Reviews*, *9*, CD010392.

**Richards et al. 2013**

Richards, J., Thorogood, M., Hillsdon, M., & Foster, C. (2013). Face-to-face versus remote and web

2.0 interventions for promoting physical activity. *The Cochrane Database of Systematic Reviews*, *9*,

CD010393.

**Short et al. 2011**

Short, C. E., James, E. L., Plotnikoff, R. C., & Girgis, A. (2011). Efficacy of tailored-print

interventions to promote physical activity: A systematic review of randomised trials. *The*

*International Journal of Behavioral Nutrition and Physical Activity*.

**Sørensen et al. 2006**

Sørensen, J. B., Skovgaard, T., & Puggaard, L. (2006). Exercise on prescription in general practice:

a systematic review. *Scandinavian Journal of Primary Health Care*, *24*(2), 69–74.

**Spana et al. 2009**

Spana, T. M., Rodrigues, R. C. M., Lourenço, L. B. D. A., Mendez, R. D. R., & Gallani, M. C. B. J.

(2009). Integrative review: behavioral interventions for physical activity practice. *Revista Latino-*

*Americana de Enfermagem*, *17*(6), 1057–1064.

**Strohacker et al. 2014**

Strohacker, K., Galarraga, O., & Williams, D. M. (2014). The impact of incentives on exercise

behavior: A systematic review of randomized controlled trials. *Annals of Behavioral Medicine*.

**Van den Berg et al. 2007**

Van den Berg, M. H., Schoones, J. W., & Vliet Vlieland, T. P. M. (2007). Internet-based physical

activity interventions: a systematic review of the literature. *Journal of Medical Internet Research*,

*9*(3), e26.

**Vandelanotte et al. 2007**

Vandelanotte, C., Spathonis, K. M., Eakin, E. G., & Owen, N. (2007). Website-delivered physical

activity interventions a review of the literature. *American Journal of Preventive Medicine*, *33*(1),

54–64.

**Whitt-Glover & Kumanyika. 2009**

Whitt-Glover, M. C., & Kumanyika, S. K. (2009). Systematic review of interventions to increase

physical activity and physical fitness in African-Americans. *American Journal of Health*

*Promotion!: AJHP*, *23*(6), S33–56.

## S3: Excluded studies and reasons for exclusion

### Reference list of excluded primary studies:

**Adachi et al. 2007**

Adachi, Y., Sato, C., Yamatsu, K., Ito, S., Adachi, K., Yamagami, T. (2007). A randomized

controlled trial on the long-term effects of a 1-month behavioral weight control program assisted by

computer tailored advice. *Behav Res Ther;* 45:459-70

**Albright et al. 2005**

Albright, C. L., Pruitt, L., Castro, C., Gonzalez, A., Woo, S., & King, A. C. (2005). Modifying

physical activity in a multiethnic sample of low-income women: one-year results from the IMPACT

(Increasing Motivation for Physical ACTivity) project. *Annals of Behavioral Medicine : A*

*Publication of the Society of Behavioral Medicine*, *30*(3), 191–200.

**Ard et al. 2000**

Ard, J., Rosati, R., Oddone, E. (2000). Culturally-sensitive weight loss program produces significant

reduction in weight, blood pressure, and cholesterol in eight weeks. *J Natl Med Assoc*;92:515–523.

**Armit et al. 2009**

Armit, C. M., Brown, W. J., Marshall, A. L., Ritchie, C. B., Trost, S. G., Green, A., & Bauman, A. E.

(2009). Randomized trial of three strategies to promote physical activity in general practice.

*Preventive Medicine*, *48*(2), 156–163.

**Baranowski et al. 1998**

Baranowski, T., Anderson, C., Carmack, C. (1998). Mediating variable framework in physical

activity interventions. How are we doing? How might we do better? *Am. J. Prev. Med.* 15, 266–297.

**Bassey et al. 1983**

Bassey, E. J., Patrick, J. M., Irving, J. M., Blecher, a., & Fentem, P. H. (1983). An unsupervised

“Aerobics” physical training programme in middle-aged factory workers: Feasibility, validation and

response. *European Journal of Applied Physiology and Occupational Physiology*, *52*(1), 120–125.

**Bauer et al 1985**

Bauer, R., Heller, R., Challah, S. (1985). United Kingdom heart disease prevention project: 12-year

follow-up of risk factors. *Am J Epidemiol;* 121(4):563–9.

**Befort et al. 2008**

Befort, C., Nollen, N., Ellerbeck, E., Sullivan, D., Thomas, J., Ahluwalia, J. (2008). Motivational

interviewing fails to improve outcomes of a behavioral weight loss program for obese African

American women: a pilot randomized trial. *J Behav Med*; 31: 367–377

**Beresford et al. 2007**

Beresford, S., Locke, E., Bishop, S., West, B., McGregor, B., Bruemmer, B., et al. (2007). Worksite

Study Promoting Activity and Changes in Eating (PACE): Design and Baseline Results. *Obesity*

*novembro*; 15(Suppl 1):4–15

**Bertozzi et al. 2004**

Bertozzi, N., Bakken, E., Bolognesi, M., Castoldi, F., Massarini, M., Palazzi, M., Pietrantoni, L.,

Righi, F., Vitali, P. (2004). Promoting physical activity in overweight and obese patients: counseling

in primary care from Italy (Cesena, 2002–2003). *Sport Sci Health*; 16: 25–30.

**Blair Irvine et al. 2004**

Blair Irvine, A., Ary, D., Grove, D., Gilfillan-Morton, L. (2004). The effectiveness of an interactive

multimedia program to influence eating habits. *Health Educ Res*;19:290-305

**Booth et al. 2008**

Booth, A. O., Nowson, C. a., & Matters, H. (2008). Evaluation of an interactive, Internet-based

weight loss program: A pilot study. *Health Education Research*, *23*(3), 371–381.

**Bull 1999**

Bull, F., Kreuter, M., Scharff, D. (1999). Effects of tailored, personalized and general health

messages on physical activity. *Patient Education & Counseling;* 36:181-192

**Burke et al. 2003**

Burke, V., Giangiulio, N., Gillam, H., Beilin, L., Houghton, S. (2003). Physical activity and nutrition

programs for couples: a randomized controlled trial1 365. *J. Clin. Epidemiol;* 56, 421–432

**Burton et al. 1995**

Burton, L., Paglia, M., German, P., et al. (1995). The effect among older persons of a general

preventive visit on three health behaviors: smoking, excessive alcohol drinking, and sedentary

lifestyle. *Prev Med*; 24:492–7

**Carels et al. 2008**

Carels, A., Konrad, K., Young, K., Darby, L., Coit, C., Clayton, A., Oemig, C. (2008). Taking

control of your personal eating and exercise environment: a weight maintenance program. *Eat*

*Behav*; 9: 228–237

**Carels et al. 2005**

Carels, R., Darby, L., Cacciapaglia, H., Douglass, O., Harper, J., Kaplar, M., Konrad, K., Rydin, S.,

Tonkin, K. (2005). Applying a stepped-care approach to the treatment of obesity. *J Psychosom Res*;

59: 375–383

**Castro et al. 1999**

Castro, C. M., Sallis, J. F., Hickmann, S. a., Lee, R. E., & Chen, A. H. (1999). A prospective study

of psychosocial correlates of physical activity for ethnic minority women. *Psychology & Health*,

*14*(2), 277–293.

**Charness & Hneezy. 2009**

Charness, G., Hneezy, U. (2009). Incentives to Exercise. *Econometrica*, *77*(3), 909–931.

**Clarke et al. 2007**

Clarke, K., Freeland-Graves, J., Klohe-Lehman, D., et al. (2007). Promotion of Physical Activity in

Low-Income Mothers Using Pedometers. *J. Am. Diet. Assoc.* 107, 962–967

**Colemant et al. 1999**

Coleman, K. J., Raynor, H. R., Mueller, D. M., Cerny, F. J., Dorn, J. M., & Epstein, L. H. (1999).

Providing sedentary adults with choices for meeting their walking goals. *Preventive Medicine*,

*28*(5), 510–519.

**Costanzo et al. 2006**

Costanzo, C., Walker, S., Yates, B., et al. (2008). Physical activity counseling for older women. *West*

*J Nurs Res*; 28(7):786–801

**Cox et al. 2003**

Cox, K., Burke, V., Gorely, T., Beilin, L., Puddey, I. (2003). Controlled comparison of retention and

adherence in home- vs center-initiated exercise interventions in women ages 40–65 years: The

S.W.E.A.T. Study (Sedentary Women Exercise Adherence Trial). *Prev. Med;* 36, 17–29

**Damschroder et al. 2010**

Damschroder, L., Lutes, L., Goodrich, D., Gillon, L., Lowery, J. (2010). A small-change approach

delivered via telephone promotes weight loss in veterans: results from the ASPIRE-VA pilot study.

*Patient Educ Couns*; 79: 262–266

**De Cocker et al. 2011**

De Cocker, K. a., De Bourdeaudhuij, I. M., Brown, W. J., & Cardon, G. M. (2011). Four-year

follow-up of the community intervention “10000 steps Ghent.” *Health Education Research*, *26*(2),

372–380.

**De Vries et al. 2008**

de Vries, H., Kremers, S., Smeets, T., Brug, J., Eijmael, K. (2008). The effectiveness of tailored

feedback and action plans in an intervention addressing multiple health behaviors. *American Journal*

*of Health Promotion*; 22:417-425

**Edmunds et al. 2007**

Edmunds, J., Ntounamis, N., Duda, J. (2007). Adherence and well being in overweight and obese

patients referred to an exercise on prescription scheme: a self-determination theory perspective.

*Psychol Sport Exerc;* 8: 722–740

**Elder et al. 2005**

Elder, J., Ayala, G., Campbell, N., et al. (2005). Interpersonal and print nutrition communication for

a Spanish-dominant Latino population: Secretos de la Buena Vida. *Health Psychol*;24:49-57

**Emmons et al. 1999**

Emmons, K., Linnan, L., Shadel, W., et al. (1999). The working healthy project: a worksite healthpromotion

trial targeting physical activity, diet and smoking. *J Occup Environ Med*;41:545–555.

**Folta et al. 2009**

Folta, S., Lichtenstein, A., Seguin, R., Goldberg, J., Kuder, J., Nelson, M. (2009). The Strong

Women-Healthy Hearts program: reducing cardiovascular disease risk factors in rural sedentary,

over- weight, and obese midlife and older women. *Am J Public Health;* 99: 1271–1277.

**Ferney et al. 2009**

Ferney, S. L., Marshall, A. L., Eakin, E. G., & Owen, N. (2009). Randomized trial of a

neighborhood environment-focused physical activity website intervention. *Preventive Medicine*,

*48*(2), 144–150.

**Fitzgibbon et al. 2005**

Fitzgibbon, M. L., Stolley, M. R., Ganschow, P., Schiffer, L., Wells, A., Simon, N., & Dyer, A.

(2005). Results of a faith-based weight loss intervention for black women. *Journal of the National*

*Medical Association*, *97*(10), 1393–1402.

**French et al. 2011**

French, S., Gerlach, A., Mitchell, N., et al. (2011). Household obesity prevention: Take Action—a

group-randomized trial. *Obesity;* 19(10):2082–2088.

**French et al. 2010**

French, S., Harnack, L., Hannan, P., Mitchell, N., Gerlach, A., & Toomey, T. (2010). Worksite

environment to prevent obesity among metropolitan transit workers. **Journal?**

**Gaston et al. 2007**

Gaston, M. H., Porter, G. K., & Thomas, V. G. (2007). Prime Time Sister Circles: evaluating a

gender-specific, culturally relevant health intervention to decrease major risk factors in mid-life

African-American women. *Journal of the National Medical Association*, *99*(4), 428–438.

**Gilson et al. 2007**

Gilson, N., McKenna, J., Cooke, C., & Brown, W. (2007). Walking towards health in a university

community: a feasibility study. *Preventive Medicine*, 44, 167-169.

**Gilson et al. 2009**

Gilson, N. D., Puig-Ribera, A., McKenna, J., Brown, W. J., Burton, N. W., & Cooke, C. B. (2009).

Do walking strategies to increase physical activity reduce reported sitting in workplaces: a

randomized control trial. *The International Journal of Behavioral Nutrition and Physical Activity*, *6*,

**Godin et al. 1987**

Godin, G., Deshamais, Jobin, J., Cook, J. (1987). The impact of physical fitness and health age

appraisal upon exercise intentions and behaviour. *J Behav Med*;10:241-50

**Gomel et al. 1993**

Gomel, M., Oldenburg, B., Simpson, J., Owen, N. (1993). Work-site cardiovascular risk reduction: a

randomized trial of health risk assessment, education, counseling, and incentives. *Am J Pub Health;*

83(9):1231–8.

**Harland et al. 1999**

Harland, J., White, M., Drinkwater, C., Chinn, D., Farr, L., & Howel, D. (1999). The Newcastle

exercise project: a randomised controlled trial of methods to promote physical activity in primary

care. *BMJ (Clinical Research Ed.)*, *319*(7213), 828–832.

**Harrison et al. 2005**

Harrison, R., Roberts, C., Elton, P. (2005). Does primary care referral to an exercise programme

increase physical activity one year later? A randomized controlled trial. *J Public Health; 27*: 25–32

**Harvey-Berino et al. 2002**

Harvey-Berino, J., Pintauro, S., Buzzell, P., et al. (2002). Does using the Internet facilitate the

maintenance of weight loss? *Int J Obes*; 26: 1254–1260

**Heirich et al. 1993**

Heirich, M. a, Foote, A., Erfurt, J. C., & Konopka, B. (1993). Work-Site Physical Fitness Programs.

*Journal of Occupational Medicine*.

**Hovell et al. 2008**

Hovell, M. F., Mulvihill, M. M., Buono, M. J., Liles, S., Schade, D. H., Washington, T. a., … Sallis,

J. F. (2008). Culturally tailored aerobic exercise intervention for low-income Latinas. *American*

*Journal of Health Promotion*, *22*(3), 155–163.

**Huang et al. 2009**

Huang, S., Hung, W., Chang, M., Chang, J. (2009). The effect of an internet-based, stage-matched

message intervention on young taiwanese women’s physical activity. *J Health Commun*; 14:210–227

**Jacobs et al. 2004**

Jacobs, A., Ammerman, A., Ennett, S., et al. (2004). Effects of a Tailored Follow-Up Intervention on

Health Behaviors, Beliefs, and Attitudes. *Journal of Women's Health;* 13:557-68

**Jeffery et al. 1998**

Jeffery, R., Wing, .R, Thorson, C., Burton, L. (1998). Use of personal trainers and financial

incentives to increase exercise in a behavioral weight-loss program. *J Consult Clin Psychol*; 66: 777-

783

**Jorna et al. 2006**

Jorna, M., Ball, K., Salmon, J. (2006). Effects of a holistic health program on women’s physical

activity and mental and spiritual health. *J Sci Med Sport;* 9(5):395–401

**Kennedy et al. 2005**

Kennedy, B., Paeratakil, S., Champagne, C., Ryan, D., Harsha, D., McGee, C., Johnson, G., Deyhim,

F., Forsythe, W., Bogle, M. (2005). Church-Based Wegith Loss Program for Africian-Amercian

Adults Using Church Members as Health Educators: A Comparision of Inidividual and Group

Intervention. *Ethnicity & Disease*, *15*.

**Kim et al. 2004**

Kim, S., Koniak-Griffin, D., Flaskerud, J.H., Guarnero, P.A. (2004). The impact of lay health

advisors on cardiovascular health promotion: using a community-based participatory approach. *J.*

*Cardiovasc. Nurs;* 19, 192–199

**King & Frederiksen. 1984**

King, A., Frederiksen, L. (1984). Low-cost strategies for increasing exercise behaviour. *Behav*

*Modif;* 8:3-2 1

**Kreuter & Strecher. 1996**

Kreuter, M., Strecher, V. (1996). Do tailored behavior change messages enhance the effectiveness of

health risk appraisal? Results from a randomized trial. *Health Education Research*; 11:97-105

**Kypri & McAnally. 2005**

Kypri, K., McAnally, H. (2005). Randomized controlled trial of a web- based primary care

intervention for multiple health risk behaviors. *Prev Med;* 41:761-766.

**Lawton et al. 2009**

Lawton, B. a, Rose, S. B., Raina Elley, C., Dowell, A. C., Fenton, A., & Moyes, S. a. (2009).

Exercise on prescription for women aged 40-74 recruited through primary care: two year

randomised controlled trial. *British Journal of Sports Medicine*, *43*(2), 120–123.

**Leermakers et al. 1999**

Leermakers, E., Perri, M., Shigaki, C., Fuller, P. (1999). Effects of exercise-focused versus weight focused maintenance programs on the management of obesity. *Addict Behav;* 24: 219–227

**Leslie et al. 2005**

Leslie, E., Marshall, A., Owen, N., Bauman, A. (2005). Engagement and retention of participants in a

physical activity website. *Prev Med;* 40:54–9.

**Lovibond et al. 1986**

Lovibond, S. H., Birrell, P. C., & Langeluddecke, P. (1986). Changing coronary heart disease risk factor status: the effects of three behavioral programs. *Journal of Behavioral Medicine*, *9*(5).

**Lombar et al. 2010**

Lombard, C., Deeks, A., Jolley, D., Ball, K., & Teede, H. (2010). A low intensity, community based

lifestyle programme to prevent weight gain in women with young children: cluster randomised

controlled trial. *BMJ (Clinical Research Ed.)*, *341*, c3215.

**Marcus et al. 2007**

Marcus, B. H., Lewis, B. a, Williams, D. M., Dunsiger, S., Jakicic, J. M., Whiteley, J. a, … Parisi,

A. F. (2007). A comparison of Internet and print-based physical activity interventions. *Archives of*

*Internal Medicine*, *167*(9), 944–949.

**Marshall et al. 2003**

Marshall, A. L., Bauman, A. E., Owen, N., Booth, M. L., Crawford, D., & Marcus, B. H. (2003).

Population-based randomized controlled trial of a stage-targeted physical activity intervention.

*Annals of Behavioral Medicine*, *25*(3), 194–202.

**Marshall et al. 2005**

Marshall, A. L., Booth, M. L., & Bauman, A. E. (2005). Promoting physical activity in Australian

general practices: A randomised trial of health promotion advice versus hypertension management.

*Patient Education and Counseling*, *56*(3), 283–290.

**Martin et al. 1984**

Martin, J., Dubbert, P., Katell, A., et al. (1984). Behavioral control of exercise in sedentary adults:

Studies 1 through 6. *J Consult Clin Psychol;* 52: 795-811

**McAuley et al. 1994**

McAuley, E., Courneya, K., Rudolph, Lox, C. (1994). Enhancing Exercise Adherence in Males and

Females. *Preventative Medicine*, *23*, 498–506.

**McConnon et al. 2007**

McConnon, A., Kirk, S., Cockroft, J., et al. (2007). The Internet for weight control in an obese

sample: Results of a randomised controlled trial. *BMC Health Serv Res*; 7: 206

**McEachan et al. 2011**

McEachan, R. R. C., Lawton, R. J., Jackson, C., Conner, M., Meads, D. M., & West, R. M. (2011).

Testing a workplace physical activity intervention: a cluster randomized controlled trial. *The*

*International Journal of Behavioral Nutrition and Physical Activity*, *8*(1), 29.

**McNabb et al. 1997**

McNabb, W., Quinn, M., Kerver, J., et al. (1997). The PATHWAYS church-based weight loss

program for urban African-American women at risk for diabetes. *Diabetes Care*; 20:1518–1523

**Miller et al. 2002**

Miller, Y., Trost, S., Brown, W. (2002). Mediators of physical activity behavior change among

women with young children. *Am J Prev Med*; 23(2 Suppl.):98–103

**Morgan et al. 2011**

Morgan, P. J., Collins, C. E., Plotnikoff, R. C., Cook, A. T., Berthon, B., Mitchell, S., & Callister, R.

(2011). Efficacy of a workplace-based weight loss program for overweight male shift workers: The

Workplace POWER (Preventing Obesity Without Eating like a Rabbit) randomized controlled trial.

*Preventive Medicine*, *52*(5), 317–325.

**Murphy et al. 2006**

Murphy, M., Murtagh, M., Boreham, C., Hare, L., & Nevill, A. (2006) The effect of a worksite based

walking programme on cardiovascular risk in previously sedentary civil servants. *BMC Public*

*Health*, 6 Article number: 136.

**Nader et al. 1989**

Nader, P., Sallis, J, Patterson, T, Abramson, I, Rupp, J., Senn, K., et al. (1989). A family approach to

cardiovascular risk reduction: Results from the San Diego family health project. *Heath Education*

*Quarterly,* 16, 229-244

**Napolitano & Marcus. 2002**

Napolitano, M., Marcus, B. (2002). Targeting and tailoring physical activity information using print

and information technologies. *Exerc Sport Sci Rev*;30(3):122–8.

**Norris et al. 2000**

Norris, S. L., Grothaus, L. C., Buchner, D. M., & Pratt, M. (2000). Effectiveness of physician-based

assessment and counseling for exercise in a staff model HMO. *Preventive Medicine*, *30*(6), 513–

523.

**Oenema et al. 2008**

Oenema, A., Brug, J., Dijkstra, A., deWeerdt, I., de Vries, H. (2008). Efficacy and use of an internet delivered computer-tailored lifestyle intervention, targeting saturated fat intake, physical activity and smok- ing cessation: A randomized controlled trial. *Ann Behav Med*; 35:125-35.

**Ostwald 1989**

Ostwald, S. (1989). Changing employees’ dietary and exercise practices: An experimental study in a

small company. *J Occup Med*; 31(2):90–7.

**Owen et al. 1987**

Owen, N., Lee, C., Naccarella, L., Haag, K. (1987). Exercise by mail: a mediated behavior-change

program for aerobic exercise*. J Sport Psychol;* 9: 346–57

**Parrott et al. 2008**

Parrott, M., Tennant, L., Olejnik, S., Poudevigne, M. (2008). Theory of planned behavior:

Implications for an email-based physical activity intervention. *Psychol Sport Exerc*; 9:511–526.

**Pereira et al. 1998**

Pereira, M. a., Kriska, A. M., Day, R. D., Cauley, J. a., LaPorte, R. E., & Kuller, L. H. (1998). A

Randomized Walking Trial in Postmenopausal Women. *Archives of Internal Medicine*, *158*(15),

1695.

**Plotnikoff et al. 2005**

Plotnikoff, R., Mccargar, L., Wilson, P., Loucaides,C. (2005). Efficacy of an E-mail intervention for

the promotion of physical activity and nutrition behavior in the workplace context. *Am J Health*

*Promot*; 19(6):422–9

**Prestwich et al. 2010**

Prestwich, A., Perugini, M., & Hurling, R. (2010). Can implementation intentions and text messages

promote brisk walking? A randomized trial. *Health Psychology : Official Journal of the Division of*

*Health Psychology, American Psychological Association*, *29*(1), 40–49.

**Pope & Harvey-Berino. 2013**

Pope, L., Harvey-Berino, J. (2013). Burn and earn: A randomized controlled trial incentivizing

exercise during fall semester for college first-year students. *Prev Med*; 56: 197-201.

**Prochaska et al. 2005**

Prochaska, J., Velicer, W., Redding, C., et al. (2005). Stage-based expert systems to guide a

population of primary care patients to quit smoking, eat healthier, prevent skin cancer, and receive

regular mammograms. *Prev Med*; 41:406-16

**Prochaska et al. 2004**

Prochaska, J., Velicer, W., Rossi, J., et al. (2004). Multiple risk expert systems interventions: Impact

of simultaneous stage-matched ex- pert system interventions for smoking, high-fat diet, and sun

expo- sure in a population of parents. *Health Psychol;* 23:503-16

**Purath et al. 2004**

Purath, J., Miller, A. M., McCabe, G., & Wilbur, J. (2004). A brief intervention to increase physical

activity in sedentary working women. *The Canadian Journal of Nursing Research = Revue*

*Canadienne de Recherche En Sciences Infirmieres*, *36*(1), 76–91.

**Quinn et al. 2008**

Quinn, A., Doody, C., O’Shea, D. (2008). The effect of a physical activity education programme on

physical activity, fitness, quality of life and attitudes to exercise in obese females. *J Sci Med Sport;*

11: 469–472.

**Quintilianni et al. 2010**

Quintiliani, L. M., Campbell, M. K., Bowling, J. M., Steck, S., Haines, P. S., & DeVellis, B. M.

(2010). Results of a randomized trial testing messages tailored to participant-selected topics among

female college students: physical activity outcomes. *Journal of Physical Activity & Health*, *7*(4),

517–526.

**Reid & Morgan, 1979**

Reid, E. L., & Morgan, R. W. (1979). Exercise prescription: a clinical trial. *American Journal of*

*Public Health*, *69*(6), 591–595.

**Richardson et al. 2005**

Richardson, C., Brown, B., Foley, S., Dial, K., Lowery, J. (2005). Feasibility of adding enhanced

pedometer feedback to nutritional counseling for weight loss. *J Med Internet Res;* 7: 56–69.

**Rovinak et al. 2005**

Rovniak, L. S., Hovell, M. F., Wojcik, J. R., Winett, R. a., & Martinez-Donate, A. P. (2005).

Enhancing theoretical fidelity: An e-mail-based walking program demonstration. *American Journal*

*of Health Promotion*, *20*(2), 85–95.

**Schelling et al. 2009**

Schelling, S., Munsch, S., Meyer, A. H., Newark, P., Biedert, E., & Margraf, J. (2009). Increasing

the motivation for physical activity in obese patients. *International Journal of Eating Disorders*,

*42*(2), 130–138.

**Sciamanna et al. 2002**

Sciamanna, C., Lewis, B., Tate, D., Napolitano, M., Fotheringham, M., Marcus, B. (2002). User

attitudes toward a physical activity promotion website. *Prev Med;* 35:612–5.

**Sidman et al. 2004**

Sidman, C., Corbin, C., Le Masurier, G. (2004). Promoting physical activity among sedentary

women using pedometers. *Res Q Exerc Sport;* 75(2):122–129

**Silva et al. 2010**

Silva, M., Vieira, P., Coutinho, S., Minderico, C., Matos, M., Sardinha, L., Teixeira, P. (2010). Using

self-determination theory to promote physical activity and weight control: a randomized controlled

trial in women. *J Behav Med;* 33: 110– 122

**Skar et al. 2011**

Skår, S., Sniehotta, F. F., Molloy, G. J., Prestwich, A., & Araújo-Soares, V. (2011). Do brief online

planning interventions increase physical activity amongst university students? A randomised

controlled trial. *Psychology & Health*, *26*(4), 399–417.

**Sjogren et al. 2006**

Sjögren, T., Nissinen, K. J., Järvenpää, S. K., Ojanen, M. T., Vanharanta, H., & Mälkiä, E. a.

(2006). Effects of a physical exercise intervention on subjective physical well-being, psychosocial

functioning and general well-being among office workers: A cluster randomized-controlled crossover

design. *Scandinavian Journal of Medicine and Science in Sports*, *16*(6), 381–390.

**Smeets et al. 2007**

Smeets, T., Kremers, S. P. J., De Vries, H., & Brug, J. (2007). Effects of tailored feedback on

multiple health behaviors. *Annals of Behavioral Medicine*, *33*(2), 117–123.

**Smeets et al. 2008**

Smeets, T., Brug, J., & De Vries, H. (2008). Effects of tailoring health messages on physical

activity. *Health Education Research*, *23*(3), 402–413.

**Smith et al. 2009**

Smith, D., Carr, L., Dorozynski, C., Gomashe, C. (2009). Internet-delivered lifestyle physical

activity intervention: Limited inflammation and antioxidant capacity efficacy in overweight adults. *J*

*Appl Physiol;* 106:49–56

**Sorensen et al. 2005**

Sorensen, G., Barbeau, E., Stoddard, A. M., Hunt, M. K., Kaphingst, K., & Wallace, L. (2005).

Promoting behavior change among working-class, multi-ethnic workers: Results of the Healthy

Directions Small Business Study. *American Journal of Public Health;* 95, 1389–1395.

**Spittaels et al. 2007**

Spittaels, H., De Bourdeaudhuij, I., Brug, J., & Vandelanotte, C. (2007). Effectiveness of an online

computer-tailored physical activity intervention in a real-life setting. *Health Education Research*,

*22*(3), 385–396

**Spittaels & Bourdeaudhuij. 2006**

Spittaels, H., De Bourdeaudhuij, I. (2006). Implementation of an online tailored physical activity

intervention for adults in Belgium. *Health Promot Int*; 21: 311–319

**Steptoe et al. 2000**

Steptoe, A., Rink, E., Phil, M., Kerry, S. (2000). Psychosocial predictors of changes in physical

activity in overweight sedentary adults following counseling in primary care. *Prev Med;* 32: 183–

194.

**Steptoe et al. 1999**

Steptoe, a, Doherty, S., Rink, E., Kerry, S., Kendrick, T., & Hilton, S. (1999). Behavioural

counselling in general practice for the promotion of healthy behaviour among adults at increased

risk of coronary heart disease: randomised trial. *BMJ (Clinical Research Ed.)*, *319*(7215), 943–947;

discussion 947–948.

**Sternfeld et al. 2009**

Sternfeld, B., Block, C., Quesenberry, C. P., Block, T. J., Husson, G., Norris, J. C., … Block, G.

(2009). Improving Diet and Physical Activity with ALIVE. A Worksite Randomized Trial.

*American Journal of Preventive Medicine*, *36*(6), 475–483.

**Stoddard et al. 2004**

Stoddard, A., Palombo, R., Troped, P., Sorensen, G., Will, J. (2004). Cardiovascular disease risk

reduction: the Massachusetts WISE- WOMAN project. *J Womens Health* (Larchmt); 13: 539– 546.

**Suter & Marti. 1992**

Suter, E., Marti, B. (1992). Predictors of exercise adoption and adherence of middle-aged sedentary

men in randomised controlled trial. *Clinical Journal of Sports Medicine;* 2:261-7.

**Tanaka et al. 2010**

Tanaka, M., Adachi, Y., Adachi, K., Sato, C. (2010). Effects of a non-face-to- face behavioral

weight-control program among Japanese over- weight males: A randomized controlled trial. *Int J*

*Behav Med*; 17:17-24

**Tate et al. 2001**

Tate, D., Wing, R., & Winett, R. (2001). Using Internet Technology to Deliver a Behavioral Weight

Loss Program. *Jama*, *285*(9), 1172–1177.

**Tate et al. 2003**

Tate, D., Jackvony, E., Wing, R. (2003). Effects of Internet behavioral counseling on weight loss in

adults at risk for type 2 diabetes: a randomized trial. *JAMA*; 289:1833–6.

**Treadwell et al. 2010**

Treadwell, H., Holden, K., Hubbard, R., et al. (2010). Addressing obesity and diabetes among

African–American men: examination of a community-based model of prevention. *J Natl Med Assoc;*

102: 794–802

**Turner-McGrivy et al. 2009**

Turner-McGrievy, G., Campbell, M., Tate, D., Truesdade, K., Bowling, M., Crosby, L. (2009).

Pounds off digitally study. A randomized podcasting weight-loss intervention. *Am J Prev Med;* 37:

263–269

**Vandelanotte et al. 2007**

Vandelanotte, C., De Bourdeaudhuij, I., & Brug, J. (2007). Two-year follow-up of sequential and

simultaneous interactive computer-tailored interventions for increasing physical activity and

decreasing fat intake. *Annals of Behavioral Medicine*, *33*(2), 213–219.

**Wanner et al. 2009**

Wanner, M., Martin-Diener, E., Braun-Fahrlander, C., Bauer, G., Martin, B. (2009). The

Effectiveness of Active-Online, an Individually Tailored Physical Activity Intervention, in a Real

Life Setting: Randomized Controlled Trial

**Wilcox et al. 2007**

Wilcox, S., Laken, M., Bopp, M. et al. (2007). Increasing physical activity among church members:

community-based participatory research. *Am J Prev Med;* 32(2):131–138.

**Wing et al. 1998**

Wing, R., Venditti, E., Jakicic, J., Polley, B., Lang, W. (1998). Lifestyle intervention in overweight

individuals with a family history of diabetes. *Diabetes Care;* 21: 350–359

**Woolf et al. 2006**

Woolf, S., Krist, A., Johnson, R., Wilson, D., Rothemich, S., Norman, G., et al. (2006). A practicesponsored

Web site to help patients pursue healthy behaviors: an ACORN study. *Ann Fam Med;*

4:148–52.

**Yancey et al. 2006**

Yancey, A. K., McCarthy, W. J., Harrison, G. G., Wong, W. K., Siegel, J. M., & Leslie, J. (2006).

Challenges in improving fitness: results of a community-based, randomized, controlled lifestyle

change intervention. *Journal of Women’s Health (2002)*, *15*(4), 412–429.

**Yanek et al. 2001**

Yanek, L., Becker, D., Moy, T., Gittelsohn, J., Koffman, D. (2001). Project Joy: faith based

cardiovascular health promotion for African American women. *Public Health Rep;* 116(Suppl 1):68–

81.

###

| Table 1: Reasons for exclusion of primary studies | |
| --- | --- |
| Adachi et al. 2007 | Reported PA outcome metric not appropriate |
| Albright et al. 2005 | Inappropriate control condition |
| Ard et al. 2000 | Reported PA outcome metric not appropriate |
| Armit. 2009 | Inappropriate control condition |
| Ball et al. 2005 | Inappropriate control condition |
| Baranowski et al. 1990 | Not a RCT |
| Bassey et al. 1983 | Inappropriate control condition |
| Bauer et al. 1985 | Reported PA outcome metric not appropriate |
| Befort et al. 2008 | BMI of participants ≥ 30 |
| Bell et al. 2001 | Not a RCT |
| Beresford et al. 2007 | Reported PA outcome metric not appropriate |
| Bertozzi et al. 2004 | Reported PA outcome metric not appropriate |
| Blair Irvine et al. 2004 | Reported PA outcome metric not appropriate |
| Bock et al. 2001 | Reported PA outcome metric not appropriate |
| Booth. 2008 | Inappropriate control condition |
| Buis et al. 2009 | Not a RCT |
| Bull et al. 1999 | Reported PA outcome metric not appropriate |
| Burke et el. 2003 | Not a PA intervention |
| Burton et al. 1995 | Reported PA outcome metric not appropriate |
| Calfas et al. 2002 | Inappropriate control condition |
| Carels et al. 2005 | BMI of participants ≥ 30 |
| Carels et al. 2008 | BMI of participants ≥ 30 |
| Carr et al. 2008 | Reported PA outcome metric not appropriate |
| Castro et al. 1999 | Inappropriate control condition |
| Charness et al. 2009 | Reported PA outcome metric not appropriate |
| Charness. 2009 | Reported PA outcome metric not appropriate |
| Clarke et al. 2007 | Not a RCT |
| Coleman et al. 1998 | Inappropriate control condition |
| Costanzo et al. 2004 | Reported PA outcome metric not appropriate |
| Cox et al. 2003 | Reported PA outcome metric not appropriate |
| Cussler et al. 2008 | Inappropriate control condition |
| Damschroder et al. 2010 | BMI of participants ≥ 30 |
| De Crocker et al. 2007 | Inappropriate control condition |
| de Vried et al. 2008 | Reported PA outcome metric not appropriate |
| De Vries et al. 2008 | Reported PA outcome metric not appropriate |
| Deforche et al. 2004 | Inappropriate population (Children) |
| Do et al. 2008 | Reported PA outcome metric not appropriate |
| Dorgo et al. 2009 | Inappropriate population (Elderly) |
| Dorgo et al. 2009 | Inappropriate population (Elderly) |
| Dorgos et al. 2011 | Inappropriate population (Elderly) |
| Duncan et al. 2005 | No appropriate control |
| Edmunds et al. 2007 | BMI of participants ≥ 30 |
| Elder et al. 2005 | Reported PA outcome metric not appropriate |
| Elliot et al. 2004 | Reported PA outcome metric not appropriate |
| Elliot et al. 2007 | Reported PA outcome metric not appropriate |
| Emmons et al. 1999 | Reported PA outcome metric not appropriate |
| Epstein et al. 1980 | Missing outcome data |
| Ferney et al. 2009 | Inappropriate control condition |
| Ferreira et al. 2005 | Not a RCT |
| Fitzgibbon et al. 2005 | Inappropriate control condition |
| Folta et al. 2009 | BMI of participants ≥ 30 |
| Fortier et al. 1999 | Inappropriate control condition |
| Fortier et al. 2007 | Missing outcome data |
| Fortier et al. 2011 | Reported PA outcome metric not appropriate |
| French et al. 2010 | BMI of participants ≥ 30 |
| French et al. 2011 | Reported PA outcome metric not appropriate |
| Gamble et al. 1993 | Missing outcome data |
| Gaston et al. 2007 | Reported PA outcome metric not appropriate |
| Gerdle et al. 1995 | Reported PA outcome metric not appropriate |
| Gilson et al. 2007 | Not a RCT |
| Gilson et al. 2009 | Inappropriate control condition |
| Godin et al. 1987 | Reported PA outcome metric not appropriate |
| Gomel et al. 1993 | Missing outcome data |
| Goodpaster et al. 2010 | BMI of participants ≥ 30 |
| Grandjean et al. 1996 | Reported PA outcome metric not appropriate |
| Greaney et al. 2008 | Inappropriate population (Elderly) |
| Gronningaeter et al. 1992 | Reported PA outcome metric not appropriate |
| Haapala et al. 2009 | Reported PA outcome metric not appropriate |
| Harland. 1999 | Reported PA outcome metric not appropriate |
| Harma et al. 1988 | Reported PA outcome metric not appropriate |
| Harrison et al. 2004 | Reported PA outcome metric not appropriate |
| Harrison. 2005 | Reported PA outcome metric not appropriate |
| Harvey-Berino et al. 2002 | Reported PA outcome metric not appropriate |
| Harvey-Berino et al. 2004 | Reported PA outcome metric not appropriate |
| Haung et al. 2009 | Not a RCT |
| Heirich et al. 1993 | Reported PA outcome metric not appropriate |
| Hovell et al. 2008 | Missing outcome data |
| Hsu et al. 2011 | Inappropriate population (Elderly) |
| Huang et al. 2009 | Not a RCT |
| Irvine et al. 2004 | Reported PA outcome metric not appropriate |
| Issacs et al. 2008 | Reported PA outcome metric not appropriate |
| Jacobs et al. 2004 | Reported PA outcome metric not appropriate |
| Jeffery. 1998 | BMI of participants ≥ 30 |
| Jimmy et al. 2005 | Not a RCT |
| Jorna et al. 2006 | Not a RCT |
| Juneau et al. 1987 | Reported PA outcome metric not appropriate |
| Keller et al. 2008 | Reported PA outcome metric not appropriate |
| Kennedy et al. 2005 | Inappropriate control condition |
| Kim et al. 2004 | Not a RCT |
| King & Frederiksen. 1984 | Reported PA outcome metric not appropriate |
| King et al. 1991 | Inappropriate population (Elderly) |
| Kloek et al. 2006 | Not a RCT |
| Kreuter et al. 1996 | Reported PA outcome metric not appropriate |
| Kypri. 2005 | Reported PA outcome metric not appropriate |
| Lamb et al. 2002 | Reported PA outcome metric not appropriate |
| Lawton 2008 | Reported PA outcome metric not appropriate |
| Lee et al. 2012 | Not a RCT |
| Leermakers et al. 1999 | Reported PA outcome metric not appropriate |
| Leslie. 2005 | Reported PA outcome metric not appropriate |
| Lewis et al. 2008 | Reported PA outcome metric not appropriate |
| Lindsay-Reid and Morgan. 1979 | Reported PA outcome metric not appropriate |
| Lombard et al. 2010 | Reported PA outcome metric not appropriate |
| Lovibond et al. 1986 | Inappropriate control condition |
| Lupton et al. 2003 | Not a RCT |
| MacKinnon et al. 2010 | Reported PA outcome metric not appropriate |
| Maffiuletti et al. 2005 | BMI of participants ≥ 30 |
| Marcus et al. 1998 | Inappropriate control condition |
| Marcus et al. 2007 | Inappropriate control condition |
| Marshall 2003 | Inappropriate control condition |
| Marshall et al. 2005 | Reported PA outcome metric not appropriate |
| Martin. 1984 | Reported PA outcome metric not appropriate |
| Maruyama et al. 2010 | Reported PA outcome metric not appropriate |
| McAuley et al. | Reported PA outcome metric not appropriate |
| McAuley et al. 1994 | Missing outcome data |
| McCarthy et al. 2007 | Reported PA outcome metric not appropriate |
| McConnon et al. 2007 | Reported PA outcome metric not appropriate |
| McEachan et al. | Inappropriate control condition |
| McEachan et al. 2011 | Missing outcome data |
| McNabb et al. 1997 | BMI of participants ≥ 30 |
| Miller et al. 2002 | Reported PA outcome metric not appropriate |
| Morey et al. 2009 | Inappropriate population (Elderly) |
| Morgan. 2011 | BMI of participants ≥ 30 |
| Murphy et al. 2006 | Reported PA outcome metric not appropriate |
| Mutrie et al. 2002 | Reported PA outcome metric not appropriate |
| Nader et al. 1989 | Reported PA outcome metric not appropriate |
| Napolitano & Marcus. 2002 | Not a RCT |
| Nemet et al. 2005 | Inappropriate population (Children) |
| Nisbeth et al. 2000 | Reported PA outcome metric not appropriate |
| Norris. 2000 | Reported PA outcome metric not appropriate |
| Oenema et al. 2008 | Reported PA outcome metric not appropriate |
| Oja et al. 1991 | Reported PA outcome metric not appropriate |
| Ornes and Randsell. 2007 | Reported PA outcome metric not appropriate |
| Ostwald. 1989 | Reported PA outcome metric not appropriate |
| Owen et al. 1987 | Reported PA outcome metric not appropriate |
| OXCHECK et al. 1995 | Reported PA outcome metric not appropriate |
| Parrott et al. 2008 | Reported PA outcome metric not appropriate |
| Patrick et al. 2001 | Inappropriate population (Children) |
| Pereira et al. 1998 | Missing outcome data |
| Pereira et al. 1998 | Reported PA outcome metric not appropriate |
| Petrella et al. 2003 | Inappropriate population (Elderly) |
| Plotnikoff et al. 2005 | Not a RCT |
| Pope. 2013 | Reported PA outcome metric not appropriate |
| Prestwich et al. 2009 | Not a RCT |
| Prestwich et al. 2010 | Reported PA outcome metric not appropriate |
| Pritchard et al. 1997 | Reported PA outcome metric not appropriate |
| Prochasaka et al. 2004 | Reported PA outcome metric not appropriate |
| Prochasaka et al. 2005 | Reported PA outcome metric not appropriate |
| Purath et al. 2004 | Missing outcome data |
| Quinn et al 2008 | BMI of participants ≥ 30 |
| Quintillani et al. 2010 | Inappropriate control condition |
| Reid et al. 1979 | Reported PA outcome data not appropriate |
| Richardson et al 2005 | Not a RCT |
| Rovniak et al. 2005 | Inappropriate control condition |
| Rush et al. 2007 | Reported PA outcome metric not appropriate |
| Schelling et al 2009 | Inappropriate control condition |
| Sciamanna. 2002 | Reported PA outcome metric not appropriate |
| Sharpe et al. 1992 | Reported PA outcome metric not appropriate |
| Sharpe et al. 2010 | Not a RCT |
| Sidman et al. 2004 | Not a RCT |
| Siegel et al. 2010 | Reported PA outcome metric not appropriate |
| Silva et al. 2010 | BMI of participants ≥ 30 |
| Simmons et al. 2004 | Not a RCT |
| Sjogren et al. 2006 | Inappropriate control condition |
| Skar et al. 2011 | Inappropriate control condition |
| Slootmaker et al. | Missing outcome data |
| Slootmaker et al.2009 | Inappropriate control condition |
| Smeets et al. 2007 | Reported PA outcome metric not appropriate |
| Smeets et al. 2008 | Reported PA outcome metric not appropriate |
| Smith et al. 2003 | Not a RCT |
| Smith et al. 2009 | BMI of participants ≥ 30 |
| Sorensen et al. 2005 | Reported PA outcome metric not appropriate |
| Speck et al. 2001 | Not a RCT |
| Spittaels et al 2006 | Not a RCT |
| Spittaels. 2007 | Inappropriate control condition |
| Staffileno et al. 2007 | Reported PA outcome metric not appropriate |
| Steptoe et al 2000 | Reported PA outcome metric not appropriate |
| Steptoe et al. 1999 | Reported PA outcome metric not appropriate |
| Steptoe et al. 1999 | Reported PA outcome metric not appropriate |
| Stoddard et al 2004 | Reported PA outcome metric not appropriate |
| Stoddard et al. 2004 | Reported PA outcome metric not appropriate |
| Strenfeld et al. | Reported PA outcome metric not appropriate |
| Strenfeld et al. 2009 | Missing outcome data |
| Suter and Marti 1992 | Reported PA outcome metric not appropriate |
| Swinburn et al. 1998 | Inappropriate control condition |
| Tan et al. 2006 | Inappropriate population (Elderly) |
| Tanaka et al. 2010 | Reported PA outcome metric not appropriate |
| Tate 2003 | BMI of participants ≥ 30 |
| Tate. 2001 | Inappropriate control condition |
| Taylor et al. 2006 | Reported PA outcome metric not appropriate |
| Teri et al. 2011 | Inappropriate population (Elderly) |
| Treadwell et al. 2010 | Reported PA outcome metric not appropriate |
| Turner-McGrievy et al. 2009 | BMI of participants ≥ 30 |
| Van Stralen et al. 2009 | Inappropriate population (Elderly) |
| Van Stralen et al. 2011 | Inappropriate population (Elderly) |
| Vandelanotte et al. 2005 | Inappropriate control condition |
| Vandelanotte et al. 2007 | Inappropriate control condition |
| Veverka et al. 2003 | Missing outcome data |
| Walker et al. 2006 | Inappropriate control condition |
| Walker et al. 2009 | Reported PA outcome metric not appropriate |
| Walker et al. 2010 | Reported PA outcome metric not appropriate |
| Wanner et al. 2009 | Inappropriate control condition |
| Wendel-Vos | Not a RCT |
| Wilcox et al. 2007 | Reported PA outcome metric not appropriate |
| Williams et al. 2004 | Not a RCT |
| Wing et al. 1996 | Not a RCT |
| Wing et al. 1998 | BMI of participants ≥ 30 |
| Woolf. 2006 | Not a RCT |
| Yancey et al. 2006 | Inappropriate control condition |
| Yanek et al. 2001 | Reported PA outcome metric not appropriate |

## S4: Post intervention Cumulative Meta-analysis:

### Table 2: Application of indicators of sufficiency and stability

| **Number added to CMA** | **Trial** | **Year** | **Cumulative effect** | **LL Confidence Interval** | **UL Confidence Interval** | **Indicator Sufficiency:**  **Fail- Safe Ratio** | **Indicator**  **Stability:**  **Cumulative Slope** |
| --- | --- | --- | --- | --- | --- | --- | --- |
| 1 | Cunningham et al. | 1987 | 0.4 | 0.13 | 0.669 | 0.145574671 | . |
| 2 | King et al. (a) | 1988 | 0.442 | 0.197 | 0.688 | 0.387853357 | 0.021 |
| 3 | King et al. (b) | 1988 | 0.432 | 0.206 | 0.658 | 0.490364894 | 0.0106 |
| 4 | Lee and White. | 1997 | 0.437 | 0.222 | 0.653 | 0.614880254 | 0.0069 |
| 5 | Loughlan and Mutrie. | 1997 | 0.308 | 0.029 | 0.587 | 0.40129485 | -0.0026 |
| 6 | Stevens et al. | 1998 | 0.442 | 0.093 | 0.791 | **2.964071467** | -0.0022 |
| 7 | Goldstein et al. | 1999 | 0.361 | 0.001 | 0.721 | Threshold attained | -0.0048 |
| 8 | Smith et al. | 2000 | 0.317 | 0.012 | 0.622 | Threshold attained | -0.0065 |
| 9 | Cook et al. | 2001 | 0.244 | -0.052 | 0.54 | Threshold attained | -0.0079 |
| 10 | Simons-Morton et al. (a) | 2001 | 0.234 | -0.022 | 0.491 | Threshold attained | -0.009 |
| 11 | Simons-Morton et al. (b) | 2001 | 0.218 | -0.014 | 0.449 | Threshold attained | -0.0096 |
| 12 | Bissmer and McAuley. | 2002 | 0.219 | 0 | 0.438 | Threshold attained | -0.01 |
| 13 | Campbell et al. | 2002 | 0.208 | 0.012 | 0.403 | Threshold attained | -0.0102 |
| 14 | Green et al. | 2002 | 0.191 | 0.007 | 0.374 | Threshold attained | -0.0103 |
| 15 | Hager et al. | 2002 | 0.159 | -0.022 | 0.34 | Threshold attained | -0.0104 |
| 16 | Hillsdon et al. | 2002 | 0.148 | -0.009 | 0.305 | Threshold attained | -0.0104 |
| 17 | Lowther et al. (a) | 2002 | 0.129 | -0.025 | 0.283 | Threshold attained | -0.0104 |
| 18 | Lowther et al. (b) | 2002 | 0.128 | -0.022 | 0.278 | Threshold attained | -0.0103 |
| 19 | Pinto et al. | 2002 | 0.125 | -0.017 | 0.267 | Threshold attained | -0.0103 |
| 20 | Elley et al. | 2003 | 0.128 | -0.002 | 0.258 | Threshold attained | -0.0101 |
| 21 | Napolitano et al. | 2003 | 0.135 | 0.007 | 0.263 | Threshold attained | -0.0099 |
| 22 | Proper et al. | 2003 | 0.147 | 0.023 | 0.271 | Threshold attained | -0.0097 |
| 23 | Aittasalo et al. | 2004 | 0.142 | 0.022 | 0.262 | Threshold attained | -0.0094 |
| 24 | Campbell et al. | 2004 | 0.147 | 0.033 | 0.262 | Threshold attained | -0.0091 |
| 25 | Fahrenwald et al. | 2004 | 0.182 | 0.06 | 0.303 | Threshold attained | -0.0088 |
| 26 | Newton and Perri. | 2004 | 0.186 | 0.065 | 0.306 | Threshold attained | -0.0085 |
| 27 | Staten et al. | 2004 | 0.179 | 0.062 | 0.296 | Threshold attained | -0.0081 |
| 28 | Peterson et al. | 2005 | 0.181 | 0.066 | 0.296 | Threshold attained | -0.0078 |
| 29 | Resnicow et al. | 2005 | 0.177 | 0.068 | 0.286 | Threshold attained | -0.0075 |
| 30 | Eiben and Lissner. | 2006 | 0.19 | 0.081 | 0.298 | Threshold attained | -0.0071 |
| 31 | Napolitano et al. | 2006 | 0.184 | 0.079 | 0.289 | Threshold attained | -0.0068 |
| 32 | Young and Stewart. | 2006 | 0.135 | -0.001 | 0.27 | Threshold attained | -0.0065 |
| 33 | Cook et al. | 2007 | 0.133 | 0.003 | 0.263 | Threshold attained | -0.0063 |
| 34 | De Jong et al. | 2007 | 0.193 | 0.047 | 0.338 | Threshold attained | -0.006 |
| 35 | Hurling et al. | 2007 | 0.243 | 0.091 | 0.394 | Threshold attained | -0.0058 |
| 36 | King et al. | 2007 | 0.248 | 0.1 | 0.397 | Threshold attained | -0.0055 |
| 37 | Kirkwood et al. | 2007 | 0.252 | 0.106 | 0.399 | Threshold attained | -0.0052 |
| 38 | Marcus et al. | 2007 | 0.254 | 0.111 | 0.398 | Threshold attained | -0.005 |
| 39 | Merom et al. | 2007 | 0.254 | 0.114 | 0.393 | Threshold attained | **-0.0047** |
| 40 | Spittaels et al. | 2007 | 0.255 | 0.119 | 0.39 | Threshold attained | Threshold attained |
| 41 | Winett et al. | 2007 | 0.25 | 0.121 | 0.379 | Threshold attained | Threshold attained |
| 42 | Baker et al. | 2008 | 0.261 | 0.133 | 0.389 | Threshold attained | Threshold attained |
| 43 | Bennett et al. | 2008 | 0.26 | 0.134 | 0.387 | Threshold attained | Threshold attained |
| 44 | Cussler et al. | 2008 | 0.251 | 0.126 | 0.375 | Threshold attained | Threshold attained |
| 45 | Dunton and Robertson. | 2008 | 0.25 | 0.127 | 0.372 | Threshold attained | Threshold attained |
| 46 | Katz et al. | 2008 | 0.244 | 0.124 | 0.364 | Threshold attained | Threshold attained |
| 47 | Keyserling et al. | 2008 | 0.241 | 0.124 | 0.359 | Threshold attained | Threshold attained |
| 48 | King et al. | 2008 | 0.247 | 0.13 | 0.364 | Threshold attained | Threshold attained |
| 49 | Kinmonth et al. | 2008 | 0.241 | 0.126 | 0.355 | Threshold attained | Threshold attained |
| 50 | Opdenacker et al. | 2008 | 0.246 | 0.133 | 0.359 | Threshold attained | Threshold attained |
| 51 | Thompson et al. | 2008 | 0.239 | 0.127 | 0.35 | Threshold attained | Threshold attained |
| 52 | Dishman et al. | 2009 | 0.24 | 0.132 | 0.348 | Threshold attained | Threshold attained |
| 53 | Morgan et al. | 2009 | 0.241 | 0.134 | 0.348 | Threshold attained | Threshold attained |
| 54 | Van Wier et al. | 2009 | 0.256 | 0.146 | 0.365 | Threshold attained | Threshold attained |
| 55 | Chang et al. | 2010 | 0.26 | 0.151 | 0.368 | Threshold attained | Threshold attained |
| 56 | Fjeldsoe et al. | 2010 | 0.264 | 0.156 | 0.371 | Threshold attained | Threshold attained |
| 57 | Buman et al. | 2011 | 0.261 | 0.154 | 0.367 | Threshold attained | Threshold attained |
| 58 | Castro et al. | 2011 | 0.264 | 0.159 | 0.369 | Threshold attained | Threshold attained |
| 59 | Grandes et al. | 2011 | 0.304 | 0.151 | 0.456 | Threshold attained | Threshold attained |
| 60 | Van Keulen et al. | 2011 | 0.298 | 0.148 | 0.447 | Threshold attained | Threshold attained |
| 61 | Van Stralen et al. | 2011 | 0.294 | 0.15 | 0.439 | Threshold attained | Threshold attained |
| 62 | Dirige et al. | 2013 | 0.288 | 0.146 | 0.431 | Threshold attained | Threshold attained |

### Figure 1. Application of indicator or of sufficiency (Failsafe Ratio)


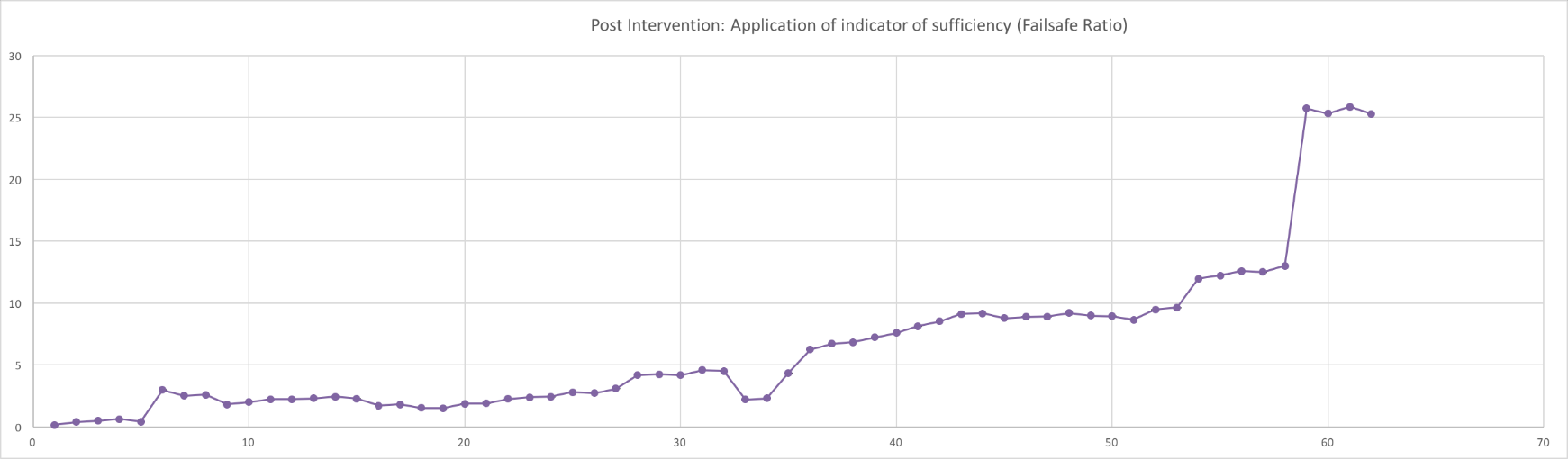


### Figure 2. Application of indicator of stability (Cumulative Slope)


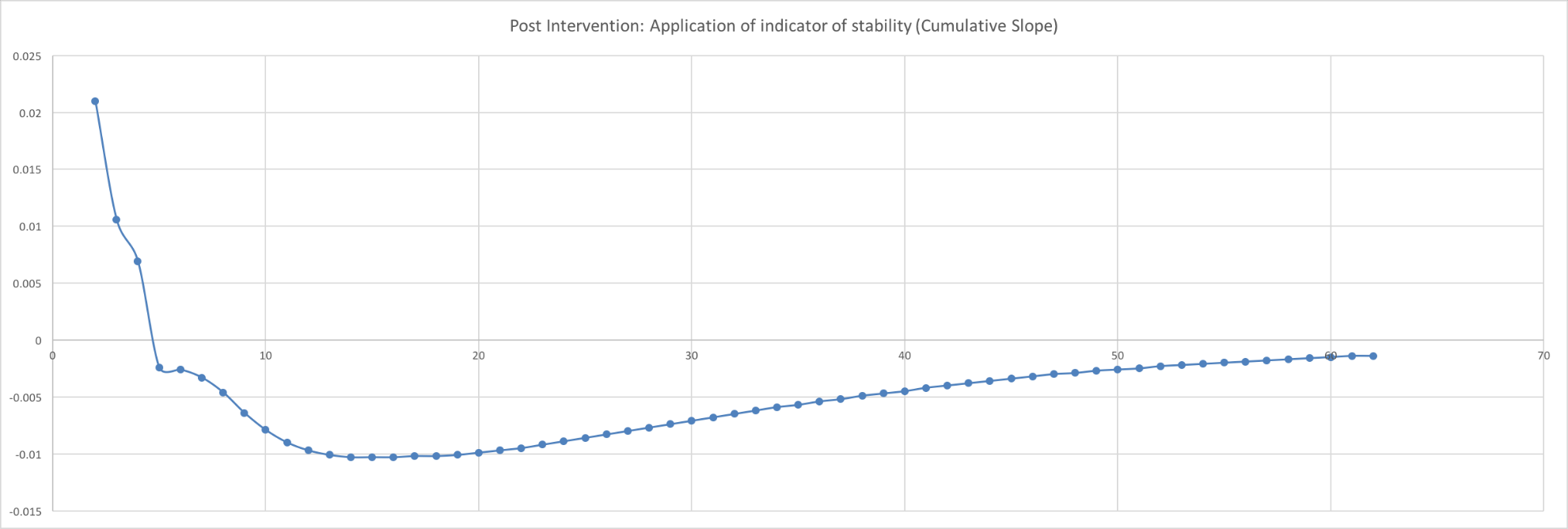


## S5: Long term follow-up Cumulative Meta-analysis

### Table 3: Application of indicators of sufficiency and stability

| **S2: Long term (12 months post baseline) CMA : Application of indicators of sufficiency and stability** | | | | | | | |
| --- | --- | --- | --- | --- | --- | --- | --- |
| **Number added to CMA** | **Trial** | **Year** | **Cumulative effect** | **LL Confidence Interval** | **UL Confidence Interval** | **Indicator Sufficiency:**  **Fail- Safe Ratio** | **Indicator**  **Stability:**  **Cumulative Slope** |
| 1 | Cunningham et al. | 1987 | 0.4 | 0.13 | 0.669 | 0.145574651 |  |
| 2 | Lee and White. | 1997 | 0.308 | -0.062 | 0.678 | 0.387853331 | -0.046 |
| 3 | Calfas et al. | 2000 | 0.219 | -0.013 | 0.451 | 0.490364885 | -0.0454 |
| 4 | Cook et al. | 2001 | 0.167 | -0.01 | 0.345 | 0.614880227 | -0.414 |
| 5 | Resnicow et al. | 2001 | 0.151 | 0.039 | 0.263 | 0.401294829 | -0.0348 |
| 6 | Simons-Morton et al. (a) | 2001 | 0.156 | 0.065 | 0.248 | **2.964071578** | -0.0265 |
| 7 | Simons-Morton et al. (b) | 2001 | 0.146 | 0.061 | 0.23 | Threshold attained | -0.0253 |
| 8 | Campbell et al. | 2002 | 0.14 | 0.064 | 0.216 | Threshold attained | -0.0224 |
| 9 | Hillsdon et al. | 2002 | 0.113 | 0.052 | 0.173 | Threshold attained | -0.0199 |
| 10 | Elley et al. | 2003 | 0.126 | 0.071 | 0.181 | Threshold attained | -0.0177 |
| 11 | Aittasalo et al. | 2004 | 0.123 | 0.069 | 0.178 | Threshold attained | -0.0158 |
| 12 | Campbell et al. | 2004 | 0.133 | 0.08 | 0.185 | Threshold attained | -0.0141 |
| 13 | Staten et al. | 2004 | 0.129 | 0.078 | 0.181 | Threshold attained | -0.0126 |
| 14 | Eiben and Lissner. | 2006 | 0.138 | 0.081 | 0.195 | Threshold attained | -0.0113 |
| 15 | King et al. | 2007 | 0.153 | 0.09 | 0.216 | Threshold attained | -0.0102 |
| 16 | Marcus et al. | 2007 | 0.162 | 0.098 | 0.225 | Threshold attained | -0.0092 |
| 17 | Cussler et al. | 2008 | 0.155 | 0.09 | 0.22 | Threshold attained | -0.0083 |
| 18 | Keyserling et al. | 2008 | 0.154 | 0.092 | 0.215 | Threshold attained | -0.0075 |
| 19 | Kinmonth et al. | 2008 | 0.146 | 0.085 | 0.206 | Threshold attained | -0.0068 |
| 20 | Napolitano et al. | 2008 | 0.141 | 0.084 | 0.199 | Threshold attained | -0.0062 |
| 21 | Thompson et al. | 2008 | 0.136 | 0.079 | 0.193 | Threshold attained | -0.0057 |
| 22 | Buman et al. | 2011 | 0.141 | 0.084 | 0.199 | Threshold attained | -0.0052 |
| 23 | Castro et al. | 2011 | 0.152 | 0.092 | 0.212 | Threshold attained | **-0.0048** |
| 24 | Grandes et al. | 2011 | 0.164 | 0.105 | 0.224 | Threshold attained | Threshold attained |
| 25 | Van Keulen et al. | 2011 | 0.158 | 0.101 | 0.214 | Threshold attained | Threshold attained |
| 26 | Van Stralen et al. | 2011 | 0.157 | 0.105 | 0.21 | Threshold attained | Threshold attained |
| 27 | Dirige et al. | 2013 | 0.148 | 0.094 | 0.201 | Threshold attained | Threshold attained |

### Figure 3. Application of indicator of sufficiency (Failsafe Ratio)


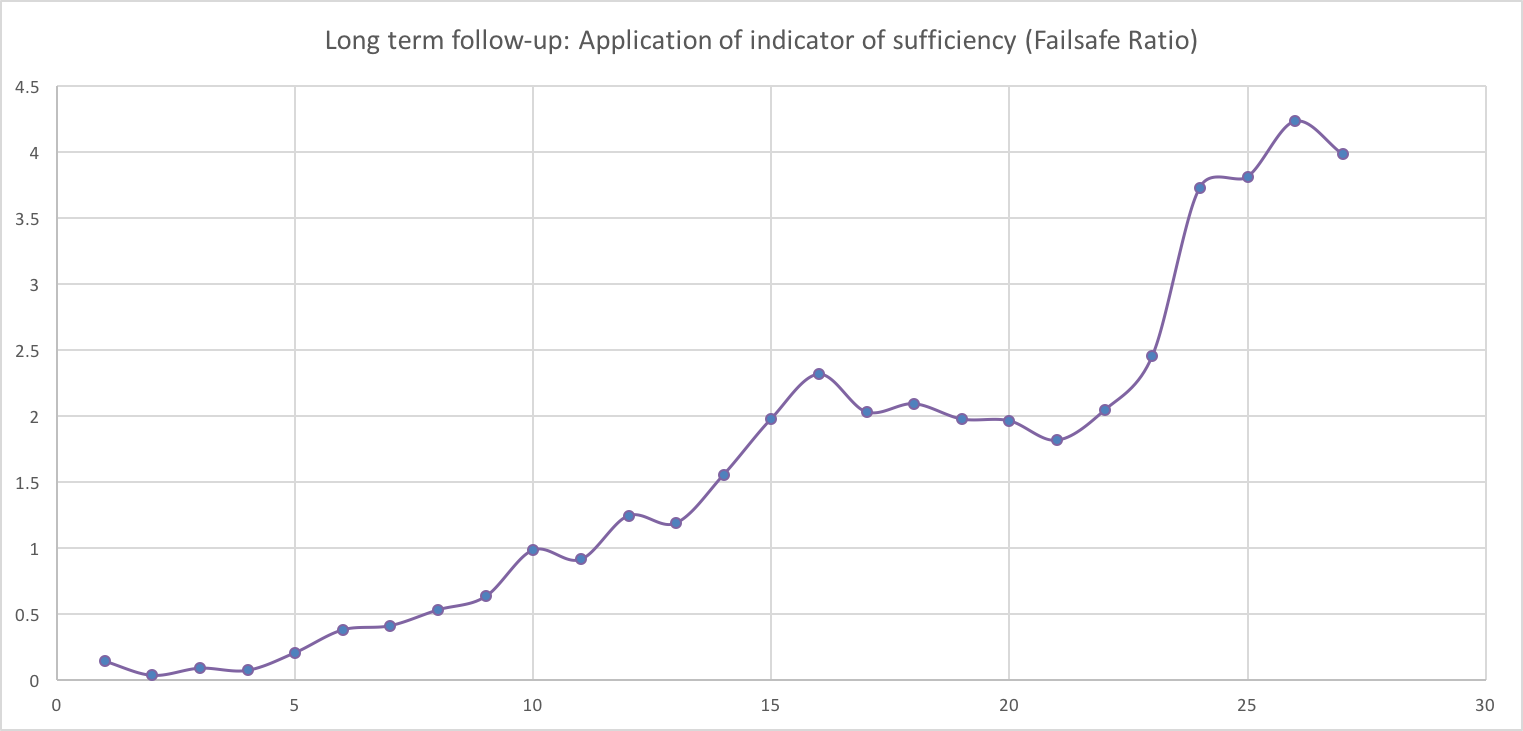


### Figure 4. Application of indicator of stability (Cumulative Slope)


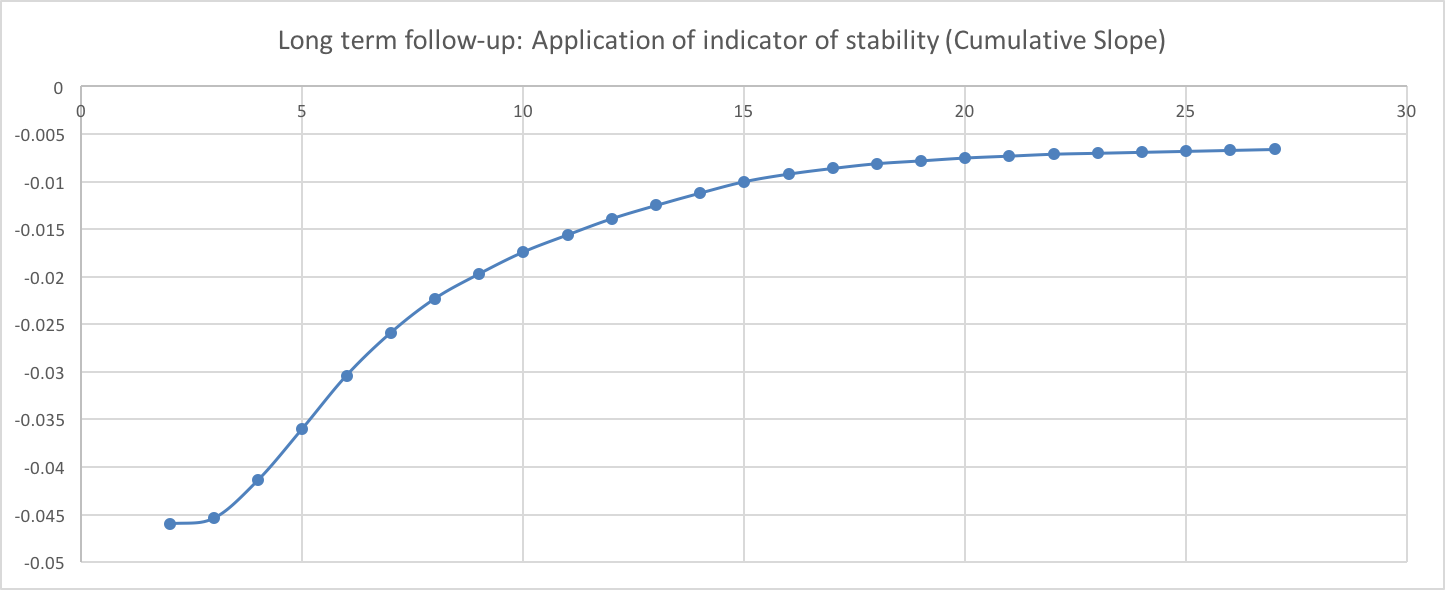

Supplement: Supplementary file 1 — Data S1: Database Search Strategies Data S2: Included primary studies and systematic reviews Data S3: Excluded studies and reasons for exclusion Data S4: Post intervention Cumulative Meta‐analysis Data S5: Long term follow‐up Cumulative Meta‐analysis Table 1: Reasons for exclusion of primary studies Table 2: Application of indicators of sufficiency and stability Table 3: Application of indicators of sufficiency and stability Figure 1. Application of indicator or of sufficiency (Failsafe Ratio) Figure 2. Application of indicator of stability (Cumulative Slope) Figure 3. Application of indicator of sufficiency (Failsafe Ratio) Figure 4. Application of indicator of stability (Cumulative Slope) [file OBR-19-1164-s001.docx]
